# Evidence-based guidelines for the use of extracorporeal membrane oxygenation in Australia and New Zealand using GRADE methodology series part 1: Venovenous extracorporeal membrane oxygenation (VV ECMO) indications and management

**DOI:** 10.1016/j.ccrj.2026.100163

**Published:** 2026-02-26

**Authors:** Sally F. Newman, Aidan Burrell, Hergen Buscher, Daniel Thomas Chung, Paul Forrest, John Fraser, Craig French, Carol Hodgson, Ryan Ruiyang Ling, Ed Litton, Graeme MacLaren, Andrew McKee, Zachary Munn, Nhi Nguyen, Julia K. Pilowski, Kollengode Ramanathan, Mark Sackley, Kiran Shekar, Myles Smith, Nikki Stamp, Madeline Wilkinson, Bruce Wilson, Priya Nair

**Affiliations:** a St Vincent's Hospital, Darlinghurst, NSW, Australia; b St Vincent's Healthcare School of Clinical Medicine, University of New South Wales, Australia; c Australian and New Zealand Intensive Care Research Centre, School of Public Health and Preventive Medicine, Monash University, VIC, Australia; d Royal Prince Alfred Hospital, Camperdown, NSW, Australia; e Critical Care Research Group, The Prince Charles Hospital & University of Queensland, Australia; f Western Health Services St Albans, VIC, Australia; g National University Hospital, Singapore; h Yong Loo Lin School of Medicine, National University of Singapore, Singapore; i Fiona Stanley Hospital, Perth, WA, Australia; j Auckland City Hospital, New Zealand; k Health Evidence Synthesis, Recommendations and Impact (HESRI), School of Public Health, The University of Adelaide, SA, Australia; l Intensive Care NSW, Agency of Clinical Innovation, Australia; m School of Public Health, Faculty of Medicine and Health, University of Sydney, Australia; n Prince Charles Hospital, Brisbane, QLD, Australia; o Te Puna Wai Ora, Dunedin Hospital, Te Whatu Ora, New Zealand; p The Alfred Hospital, Melbourne, VIC, Australia; q Cairns and Hinterland Hospital and Health Services District, QLD, Australia; r Tweed Valley Hospital, Cudgen, NSW, Australia; s Royal Australian Navy (retired), Sydney, NSW, Australia

**Keywords:** Venovenous extracorporeal membrane oxygenation, Acute respiratory distress syndrome, Respiratory failure, GRADE methodology, Clinical practice guidelines

## Abstract

We aim to provide evidence-based clinical practice guidelines for the use of VV ECMO in adult patients across Australia and New Zealand. Developed by a multidisciplinary panel of clinicians from both countries, alongside a methodologist and patient representatives, these guidelines were produced in accordance with the National Health and Medical Research Council (NHMRC) 2016 standards for guidelines. The GRADE (Grading of Recommendations, Assessment, Development and Evaluation) approach was used to assess the certainty of evidence and inform the development of recommendations. These guidelines are intended to support, not replace, clinical judgement and should be applied in the context of individual patient circumstances, values and preferences. In this part one of a three-part series, the Guideline Development Group (GDG) addressed four core clinical questions regarding the indications for and management of VV ECMO in adults. In addition to evidence-based recommendations, this guideline incorporates expert consensus through a well-established process and highlights important gaps in current evidence to guide future research priorities.

## 1 Introduction

VV ECMO is an increasingly used form of organ support for refractory respiratory failure. It uses technology adapted from cardiopulmonary bypass to provide gas exchange as a rescue therapy.^1^ Its use may facilitate reduced exposure to high-pressure, high-oxygen mechanical ventilation (MV), thereby optimising lung rest and minimising ventilator-induced lung injury (VILI). In selected patients with reversible lung injury, VV ECMO use may therefore improve mortality and other outcomes.^2^

The rapid increase in ECMO use and publications related to it have further fuelled interest in its use.^3^ However, rapidly expanding literature, mainly in observational studies, poses challenges for clinicians seeking to evaluate and synthesise evidence to guide practice.^4^ With studies varying in quality, methodologies and outcomes, interpretation requires critical appraisal skills and a nuanced understanding of the clinical and technical aspects of ECMO. Despite increasing use, considerable variability remains in the application of VV ECMO across centres, reflecting ongoing uncertainty about optimal patient selection, timing and supportive management strategies.^5^ Given the resource intensity, risks and potential long-term sequelae associated with VV ECMO, the development of evidence-based guidance is essential to support clinical decision-making and promote consistent, high-quality care.

The initiative was originally commissioned by the Centre for Research Excellence for Advanced Cardio-respiratory Therapies Improving Organ Support (ACTIONS-CRE)^6^ at the University of Queensland, and continues with ongoing support from the Centre for Research Excellence in Intensive Care (CRE-ICU)^7^ based at Monash University. Both CREs are federally funded by the NHMRC and are recognised as national leaders in generating high-quality, clinically relevant research in critical care. This collaboration reflects a sustained commitment to improving evidence-based practice for patients requiring advanced organ support, such as ECMO, and provides the necessary infrastructure and methodological expertise to support guideline development.

These recommendations aim to support clinicians, health services and policymakers in delivering equitable, safe and evidence-based care to critically ill patients across Australia and New Zealand.

## 2 Methods

The previously published protocol provides a detailed description of the methodology used,^8^ drawing from the NHMRC Guidelines for Guidelines framework.^9^ GRADE methodology was used,^10^ illustrating a transparent and well-established method for rating the certainty of evidence. Four questions were identified for the use of VV ECMO (Table 1). The protocol also details the pre-specified processes for consensus development and voting on recommendations. A detailed description of the methodology for each question is reported in the Supplementary Materials.

**Table 1:**
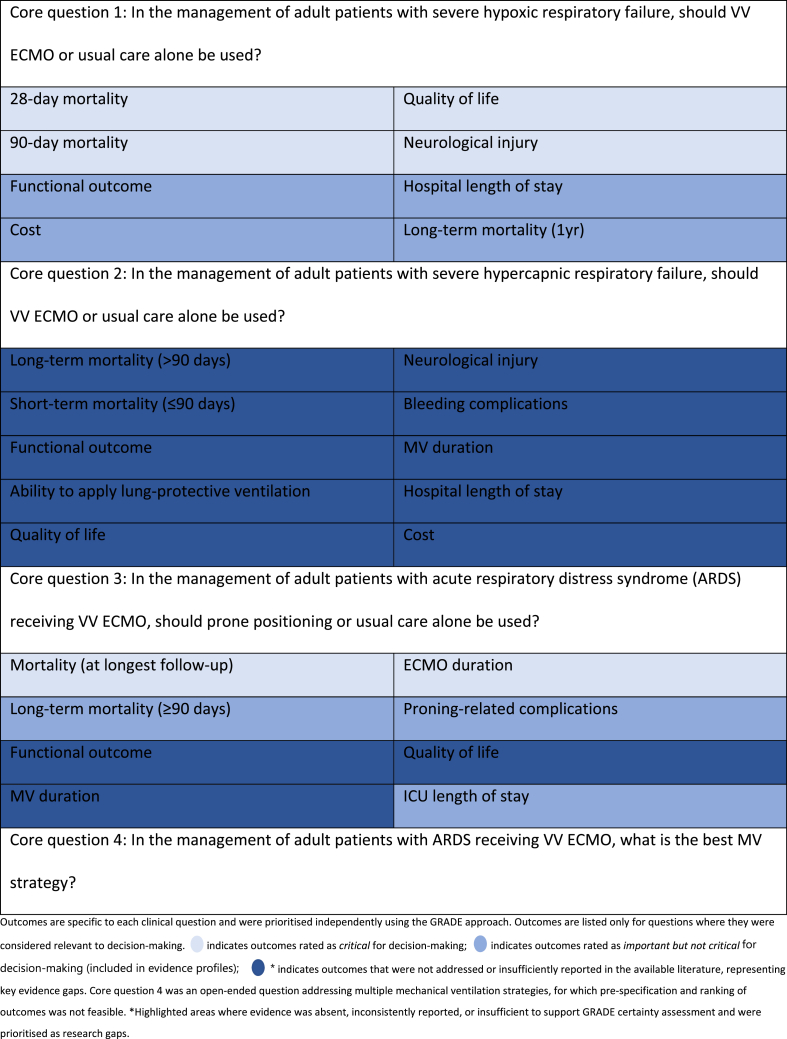
Summary of core clinical questions and outcomes.

### 2.1 Conflicts of interest

All members of the GDG completed conflict of interest declarations in accordance with NHMRC standards. Declared interests were reviewed and managed by the project leadership group, and no conflicts were identified that were considered to influence guideline development or voting. Full declarations are provided in the Supplementary Materials.

This research was produced in whole or part by UNSW Sydney researchers and is subject to the UNSW Intellectual Property policy. For the purposes of Open Access, the author has applied a “Creative Commons Attribution CC-BY licence to any Author Accepted Manuscript (AAM) version arising from this submission”.

## 3 Question 1: in the management of adult patients with severe hypoxic respiratory failure, should VV ECMO or usual care alone be used?

### 3.1 Background

Severe hypoxic respiratory failure is most commonly described and studied in terms of ARDS,^11^^,^^12^ for which there are accepted criteria for diagnosis and severity (Table 2).^13^ This guideline focuses on patients with severe ARDS who are considered for VV ECMO following failure of conventional support, defined as persistent, severe hypoxaemia despite optimal implementation of evidence-based strategies for ARDS management, including low tidal volume ventilation, application of appropriate levels of Positive End Expiratory Pressure (PEEP), high inspired oxygen concentration and the use of conjunctive measures such as prone positioning and neuromuscular blockade.

**Table 2:** ARDS defined according to the Berlin definition.^14^

Criterion	Definition
Timing	Acute onset of respiratory failure
Imaging	Bilateral lung infiltrates on CXR or CT (non-cardiac)
Oxygenation	**Mild:** PaO_2_:FiO_2_ 201–300 mmHg
	**Moderate:** 101–200 mmHg
	**Severe:** ≤100 mmHg

PaO2:FiO2 ≤300 mmHg or SpO2:FiO2 ≤315 mmHg when SpO2 ≤97 % and intubated, receiving CPAP/NIV with at least 5 cm H2O end-expiratory pressure, or high-flow nasal oxygen of ≥30L/min; CPAP- continuous positive airway pressure; NIV- non-invasive ventilation.

Contemporary studies still report high mortality for ARDS, approximately 40 % in LUNG SAFE.^15^ Severe hypoxic respiratory failure has significant morbidity, causing persistent physical and psychological sequelae.^11^^,^^12^ Therefore, appropriate management strategies are of high priority.

VV ECMO provides gas exchange and may facilitate lung-protective ventilation.^16^ It is used increasingly for severe hypoxic respiratory failure following the publication of the CESAR^16^ trial in 2009 and EOLIA^17^ 2018, and the 2009 influenza A (H1N1) and COVID-19 pandemics.^18^ However, evidence for its long-term outcomes remains unclear in adult patients.^16^^,^^19^

ECMO is an invasive, resource-intensive therapy with significant short and long-term risks such as major bleeding, renal failure, neurologic injury and long-term functional impairment. For example, in the large prospective, multicentre cohort study across 23 Australian ICUs, two-thirds of surviving patients reported moderate-to-severe disability at six months after ECMO initiation.^20^ Specifically, among patients who received VV ECMO, 54 % were either deceased or had moderate-to-severe disability at six months. New physical, psychological or cognitive disability is common, with 25 % of survivors unable to return to work.^20^^,^^21^ However, it is important to recognise that many adverse outcomes observed in ECMO populations reflect the severity of the underlying respiratory and multi-organ failure rather than being direct complications of ECMO itself.

### 3.2 Summary of the evidence

Four guidelines addressed the use of VV ECMO in severe hypoxic respiratory failure. These were published prior to one or both of the significant randomised controlled trials (RCTs)^16^^,^^17^ on this topic. They were deemed not current and not suitable for inclusion. Three systematic reviews (SRs) were found to address most critical outcomes specified (28-day mortality, 90-day mortality, hospital length of stay, quality of life and functional outcome).^2^^,^^22^^,^^23^ Primary articles included were assessed as having a low risk of bias via the relevant assessment tools.^24^^,^^25^ A search for primary literature was required for neurological injury and cost outcomes, not being adequately covered in the SRs. Two RCTs^16^^,^^17^ provided the highest quality of evidence to review these outcomes, with a low risk of bias. (Supplementary File pages 6–12).

Among 429 patients included in two trials, VV ECMO for severe hypoxic respiratory failure showed a short-term survival benefit, with a reduction in mortality at 28 days (RR 0.57 [95 % CI 0.43 to 0.76]),^2^ and 90 days (RR 0.78 [95 % CI 0.61 to 1.01]), both supported by a moderate level of certainty.^2^ Evidence for neurological injury (defined by haemorrhagic stroke), was informed by a single SR by Wang et al.,^23^ which reported an absolute risk difference (−3.9 [95 % CI -10.0 to +1.3]).^17^ Certainty was rated as moderate, although estimates were imprecise and confidence intervals crossed thresholds for clinical decision-making. Health-related quality of life (HRQoL), measured using the SF-36 questionnaire (MD 5.40 [95 % CI 4.11 to 6.68]),^22^ also showed a potential benefit with VV ECMO, although with a low level of certainty. The SF-36 is a validated instrument assessing patient-reported physical and mental health across multiple domains, scored on a scale from 0 to 100, where higher scores indicate better HRQoL.^26^ While evidence contributing to important outcomes suggests a long-term survival benefit of VV ECMO in severe hypoxic respiratory failure, with reduced mortality at one year (OR 0.48 [95 % CI 0.27 to 0.83]),^23^ the level of certainty is very low.

VV ECMO is likely associated with an acceptable cost per Quality Adjusted Life Year (QALY) (AUD 36,258 [95 % CI 13,956 to 108,401]),^16^ based on low certainty evidence. There was evidence of moderate certainty indicating that VV ECMO increased hospital length of stay in these patients (MD 14.05 days [95 % CI 8.59 to 19.52]).^2^ There was no statistically significant difference in functional outcomes, as measured by spirometry (FEV1, FVC % of predicted) between patients receiving VV ECMO and those receiving usual care alone (MD -2.58 % [95 % CI -15.59 to 10.43], MD -0.41 % [95 % CI -1.30 to 0.48]), respectively, very low certainty.^22^ Depression and anxiety as measured by Hospital Anxiety and Depression Scale (HADS) (MD -1.60 [95 % CI -1.80 to −1.39], MD -1.30 [95 % CI -2.01 to −0.59]),^22^ as well as physical and mental disability as measured by SF-36 physical component summary (PCS) and mental component summary (MCS) scores (MD 3.13 [95 % CI 2.44 to 3.83], MD 2.34 [95 % CI 1.72 to 2.96]),^22^ suggest that disability may have been reduced in survivors, with low certainty (see Supplementary Materials).

**Recommendation 1**

In adult patients with severe hypoxic respiratory failure who fulfil EOLIA criteria, we suggest the use of VV ECMO in addition to standard care.

*Conditional recommendation, very low certainty of evidence due to very low certainty of evidence in critical outcomes other than short-term mortality.*

### 3.3 Expert opinion on clinical application

While there is moderate-certainty evidence from two RCTs^16^^,^^17^ indicating short-term (28-day, 90-day) survival benefit with VV ECMO, the overall certainty of evidence was downgraded to very low due to health-related quality of life and neurological injury (reflecting the burden of disability among survivors), being rated equally critical alongside short-term mortality.

Evidence for important, but not critical for decision-making outcomes such as long-term mortality, was downgraded due to a serious risk of bias, while evidence for functional outcomes (FEV1, FVC) was downgraded due to imprecision as wide confidence intervals cross the line of no effect and inconsistency related to substantial statistical heterogeneity. Although no evidence of harm was identified that would negate the short-term benefit when considering longer-term outcomes or HRQoL, these limitations reduced overall confidence in the effect estimates. Cost-effectiveness estimates derived from the CESAR trial^16^ suggest VV ECMO is likely associated with an acceptable cost per QALY. These findings are supported by an Australian cost-utility analysis, which reported an incremental cost-effectiveness ratio of AUD 36,627 per QALY gained and AUD 18,757 per life year gained, based on a lifetime horizon from the healthcare system perspective. While these findings fall within commonly accepted willingness-to-pay thresholds, their generalisability across Australian and New Zealand health systems remains context-dependent. Differences in ECMO service configuration, including centralised versus site-based models, interhospital retrieval practices, case volume, staffing and resource utilisation, may substantially influence cost effectiveness. These factors should be considered when applying economic estimates to local policy and service planning. Therefore, the GDG issued a conditional recommendation in favour of VV ECMO. This was conditional as opposed to strong due to low or very low certainty for some of the critical outcomes.

We suggest the following criteria, adapted from those used in the EOLIA trial, when considering VV ECMO in addition to standard care for patients with severe hypoxic respiratory failure who meet diagnostic criteria for ARDS.^14^ (Table 3).

**Table 3:** Selection criteria for consideration of VV ECMO in severe hypoxic respiratory failure.

Prerequisite criteria (all must be met)	
**Domain**	**Criterion**
Airway and ventilation	Endotracheally intubated and receiving ventilation for <7 days
Optimisation of ventilation	FiO_2_ ≥80 %, Vt 6 mL per kg PBW and PEEP ≥10 cm H_2_O
Adjunctive therapies	Trial of prone positioning and neuromuscular blockade

**Physiological criteria (at least one must be met)**	

PaO_2_:FiO_2_ < 50 mmHg for > 3 h despite optimisation of MV	
PaO_2_:FiO_2_ <80 mmHg for >6 h despite optimisation of MV	
Arterial blood pH < 7.25 with partial pressure of carbon dioxide (PaCO_2_) ≥ 60 mmHg for > 6 h (respiratory rate increased to 35/min) resulting from lung protective ventilation (LPV)	

FiO2: fraction of inspired oxygen; tidal volume Vt; PBW predicted body weight.

In the individual patient data meta-analysis (MA) of two RCTs,^2^ the effect of VV ECMO on the predicted probability of ICU mortality differed by baseline organ failure burden. Patients with greater than two organ failures at randomisation showed no statistically significant mortality benefit (RR 1.00, [95 % CI 0.78 to 1.30]) whereas those with two or fewer organ failures had a substantially lower predicted ICU mortality with VV ECMO (RR 0.53, [95 % CI 0.36 to 0.78]).^2^ Interpretation may be biased by confounders, as the larger of the two trials had a significant crossover to VV ECMO as ’rescue therapy’, and multiorgan failure may reflect disease progression and the likelihood of receiving rescue therapy. Given that severe hypoxic respiratory failure may result from a wide range of underlying pathologies, significant variations in response to VV ECMO therapy in different subgroups may exist. Available evidence on subgroups is of a lesser quantity and quality than for aggregated patients with severe respiratory failure.

The COVID-19 pandemic presented a large, relatively homogeneous group of patients with respiratory failure. In the absence of high-quality RCTs, case series and cohort data have been relied upon and have shown similar outcomes to those reported in the intervention arm of published RCTs and to observational studies in non-COVID populations. In a large French registry study^27^ of COVID-19 patients treated with VV ECMO, there was a 60-day mortality of 31 %. While analysis of the 2021 ELSO Registry reported a hospital mortality of 36.9 % among patients with COVID-19 receiving VV ECMO at early adopting centres, with outcomes worsening over time to 51.9 % at late adopting centres.^28^ On this basis, we do not alter our recommendation for the use of VV ECMO in this subgroup. However, we recommend giving special consideration to resource allocation issues in the use of VV ECMO, where healthcare resources are likely to be limited.

Evidence for the use of VV ECMO in pregnant and peri-partum patients is limited by small case numbers, heterogeneity of underlying conditions, and physiological complexities such as altered haemodynamics and the need for concurrent maternal and neonatal care. Observational studies and case reports suggest favourable maternal and foetal outcomes, supported by systematic review data showing 80 % maternal and 65 % foetal survival across ECMO modalities,^29^ and registry data reporting 76.1 % maternal survival on VV ECMO.^30^ However, potential publication bias limits the certainty of these findings. These data do not justify altering the conditional recommendation for VV ECMO in this subgroup. Special considerations remain essential, particularly for foetal monitoring, anticoagulation, pharmacotherapy and delivery planning.

### 3.4 Unresolved questions and research gaps

Like other conditions associated with a high mortality, randomised trials of life-support techniques such as ECMO are associated with unique ethical and logistical problems. It is unlikely that another large RCT of ECMO in patients with severe hypoxic respiratory failure (not responding to conventional care) will be performed. However, research into mortality risk prediction will help clinicians identify patients who may benefit from the intervention. Additionally, it remains unknown if a wider and potentially earlier use of ECMO in patients with moderate disease severity will benefit a population with a lower mortality risk, a question being addressed in the REDEEM trial (NCT05562505).

## 4 Question 2: in the management of adult patients with severe hypercapnic respiratory failure, should VV ECMO or usual care be used?

### 4.1 Background

The most common clinical contexts where VV ECMO might be considered for severe hypercapnic respiratory failure without ARDS include acute severe asthma and exacerbation of chronic obstructive pulmonary disease (COPD). Hypercapnia in ARDS is usually associated with hypoxaemia and is therefore covered above. Asthma remains common, and the risk of death in severe asthma requiring invasive ventilation has been estimated at 7–10 %.^31^^,^^32^ The prevalence and morbidity of COPD is increasing globally, with the World Health Organization ranking it as the fourth leading cause of death worldwide, causing 3.23 million deaths in 2019.^33^ We acknowledge that several low-flow extracorporeal devices are available and in use for hypercapnic respiratory failure, which have been variably studied. These devices differ from high-flow VV ECMO and have not been included here.

### 4.2 Summary of available evidence

We found no RCTs that compared VV ECMO to usual care for severe hypercapnic respiratory failure (see Supplementary Materials). A single retrospective observational study^34^ examined ECMO utilisation in a large multicentre cohort of patients (encompassing 25 % of US hospital admissions from 2010 to 2020) with asthma exacerbations and respiratory failure requiring MV. All ECMO modalities were included, and no subgroup analysis or stratification by ECMO modality was available. 13,714 patients with asthma exacerbations were analysed, including 127 patients receiving ECMO, ECMO was associated with reduced mortality in the covariate-adjusted (OR 0.33 [95 % CI 0.17–0.64]), propensity score-adjusted (OR 0.36 [95 % CI 0.16–0.81]), and propensity score-matched models (OR 0.48 [95 % CI 0.24–0.98]) when compared to usual care alone.^34^ However, it must be highlighted that these results include all ECMO modalities (venovenous ECMO, veno-arterial ECMO, veno-arterial and venous ECMO).

Survival in the near-fatal asthma cohort treated with VV ECMO is notably higher compared with unmatched patients receiving VV ECMO for other indications.^35^ A single retrospective observational cohort study^36^ examined all patients receiving VV ECMO in the United Kingdom National ECMO Service from 2011 to 2017. Unpublished data provided by those authors suggest patients with asthma receiving ECMO had a numerically reduced ICU mortality (4.6 % vs 28 %) and shorter duration of ECMO (186 vs 302 h) compared to those receiving ECMO for non-asthma indications.^36^

We found insufficient quality of observational data for systematic review and therefore were unable to make a recommendation for or against the use of VV ECMO for severe hypercapnic respiratory failure.

**Recommendation 2**

We are unable to make a recommendation for or against the use of VV ECMO for severe hypercapnic respiratory failure without ARDS.

*No recommendation, no direct evidence*.

### 4.3 Expert opinion on clinical application

Based on expert opinion, VV ECMO may be considered on a case-by-case basis for patients with near-fatal asthma who fail conventional therapy. In patients with severe COPD, the evidence base is even more limited, and the use of VV ECMO should be reserved for highly selected individuals.

### 4.4 Unresolved questions and research gaps

Further research is needed to evaluate the effectiveness of VV ECMO for hypercapnic respiratory failure compared to conventional care. Future studies should use well designed matched cohort analysis to ensure comparability, accounting for variables such as disease severity, comorbidities and prior interventions. In particular, evidence addressing long-term mortality, HRQoL and functional outcomes remains limited and inconsistently reported. Future research should prioritise standardised reporting of patient-centred outcomes and complications relevant to hypercapnic respiratory failure, evaluate outcomes in specific populations, such as near-fatal asthma and COPD, to determine whether certain subgroups benefit more from ECMO should be included.

## 5 Question 3: in the management of adult patients with ARDS receiving VV ECMO, should prone positioning or usual care alone be used?

### 5.1 Background

Prone positioning has become an established intervention and is strongly recommended for patients with moderate to severe ARDS, as defined previously.^14^ Multiple RCTs, most notably the PROSEVA trial^37^ have demonstrated that early application of prolonged prone sessions (≥16 h per day) significantly improves oxygenation and reduces mortality by enhancing ventilation-perfusion matching, promoting alveolar recruitment and decreasing VILI. There has been growing interest in extending this approach to patients supported with VV ECMO given the proven physiological and survival benefits in conventionally ventilated patients.

### 5.2 Summary of available evidence

Given the recent completion of the RCT PRONECMO,^38^ the evidence team for this PICO extended the published MA by Poon et al., completed in 2021.^39^ We based the analysis on nine non-randomised and one randomised trial.^38^^,^[40], [41], [42], [43], [44], [45], [46], [47], [48], [49] A MA was conducted for the primary outcome of mortality at the longest follow-up as well as secondary outcomes including short and long-term survival, complications of ECMO, ECMO duration, and length of ICU and hospital stay (see Supplementary Materials).

Of 1448 patients, 698 received prone positioning, and 750 received standard care. The pooled analysis assessing mortality at the longest follow-up demonstrated that prone positioning, compared to usual care during VV ECMO for ARDS, was associated with a reduction in mortality (RR 0.86 [95 % CI 0.78 to 0.95]). However, it is important to note that the only RCT included in this MA (PRONECMO) did not demonstrate a significant mortality benefit, with results suggesting no difference between groups. Thus, the observed effect appears to be primarily driven by non-randomised studies.

The certainty of this effect was tempered by moderate heterogeneity, driven by serious concerns regarding time-varying confounders (such as disease severity at time of prone positioning and the timing of ECMO initiation). Additionally, the non-random allocation of the intervention, despite statistical adjustment, introduced potential selection bias and residual confounding, particularly within non-randomised cohort studies and case series, which contributed to concerns about the internal validity of the findings.

Long-term mortality assessed beyond 90 days demonstrated a statistically significant reduction in the prone positioning group compared to usual care during VV ECMO (RR 0.71 [95 % CI 0.53 to 0.94]). ECMO duration was significantly longer in the usual care group (MD 7.1 days [95 % CI 3.2 to 10.9]); however, considerable variability exists among these studies. The ICU length of stay shows a lack of strong evidence for a consistent benefit of prone positioning, high heterogeneity (I^2^ = 91 %) (MD 7.6 days [95 % CI 0.9 to 14.4]).

A trial sequential analysis (TSA) completed using propensity score matched studies and RCTs, suggested that there may be an association between prone positioning and survival that showed the cumulative association between prone positioning and mortality benefit crosses the conventional boundary (p < 0.05), but does not cross the TSA-adjusted boundary for benefit, which may or may not have been demonstrated with a larger sample size.

**Recommendation 3**

Conditional recommendation against the routine use of prone positioning for ARDS patients receiving VV ECMO.

*Conditional recommendation, very low evidence of benefit*.

### 5.3 Expert opinion on clinical application

Although the extended MA showed the potential benefit of prone positioning during VV ECMO for the ARDS population, the individual findings of the PRONECMO trial do not demonstrate a benefit.^38^ Notably, the majority of PRONECMO participants (93.5 %) had COVID-19-related ARDS, which may limit the generalisability of these findings to patients with severe non-COVID-19-related ARDS. Additionally, uncertainty remains regarding the potential undesirable effects of prone positioning during VV ECMO, given the overall low number of cases studied.

From a practical perspective, prone positioning during VV ECMO is technically complex and resource-intensive, requiring experienced multidisciplinary teams, careful consideration and adequate staffing. Potential risks include cannula displacement, bleeding and circuit complications. These feasibility, safety and resource considerations were taken into account by the GDG when formulating the conditional recommendation against routine prone positioning during VV ECMO.

### 5.4 Unresolved questions and research gaps

The only RCT was conducted during the COVID-19 pandemic, and it remains uncertain whether its findings are generalisable to non-COVID-related ARDS. Future research should focus on diverse populations and heterogeneous aetiologies of ARDS. Additionally, larger sample sizes are required to provide more reliable and statistically valid conclusions about the efficacy of prone positioning during VV ECMO.

In addition, evidence addressing outcomes beyond mortality remains limited. Data on duration of mechanical ventilation, functional outcomes and HRQoL were sparse or inconsistently reported, precluding meaningful synthesis and limiting assessment of patient-centred benefit. Larger sample sizes and standardised outcome reporting are required to provide more reliable and statistically valid conclusions regarding the efficacy and broader impact of prone positioning during VV ECMO.

## 6 Question 4: in the management of adult patients with ARDS receiving VV ECMO, what is the best mechanical ventilation strategy?

### 6.1 Background

While VV ECMO allows gas exchange in the presence of severely injured lungs and therefore may allow better organ support, it does not intrinsically contribute to lung recovery. Indeed, benefit from VV ECMO may result from facilitation of “lung rest”, as the injured lung remains susceptible to VILI. Patients with severe ARDS supported by VV ECMO often have profoundly inflamed and non-compliant lungs, rendering them highly susceptible to excessive alveolar stretch (volutrauma), high airway pressures (barotrauma) and repetitive opening and closing of unstable alveoli (atelectotrauma).^14^ Early trials of ECMO for ARDS did not routinely incorporate strategies to facilitate LPV. Since the CESAR trial, MV strategies on ECMO have improved, with greater emphasis on limiting tidal volume, plateau pressure and driving pressure that may represent an important mechanism of benefit.^50^ Therefore, choice of ventilation strategy while on ECMO is an important question for maximising the benefit from ECMO while minimising VILI. In particular, ECMO enables ultra-protective ventilation at lower or even zero tidal volume, but the optimum settings are unclear.

### 6.2 Summary of available evidence

A literature review was undertaken considering possible ventilation strategies in VV ECMO, which demonstrated themes of research interest in prevention of VILI in general (Table 4). The GRADE multi-comparison process was used,^51^ but no systematic reviews or comparative studies that evaluated competing strategies across the required range of critical outcomes were identified.

**Table 4:** MV modalities investigated for core question 4.

Ultra-low tidal volume ventilation (≤4 mL/kg)
Minimising driving pressure
High PEEP (“open lung”)
APRV
Minimising mechanical power

The literature searches identified only one systematic review that specifically addressed ventilatory parameters associated with outcomes in this population (see Supplementary Materials).^52^ This review demonstrated associations between lower driving pressures and mechanical power with improved survival, reinforcing the importance of minimising VILI during VV ECMO.^52^ However, it only assessed mortality and did not include data from the largest and most recent RCT (EOLIA). In this study, a pooled individual patient data MA used data from ten international ECMO centres involving 545 adult patients with ARDS treated with VV ECMO.^52^ The study did not compare predefined ventilation strategies; rather, it analysed the associations between specific ventilator settings during the first three days of ECMO support and hospital mortality. The only ventilatory parameter found to be independently associated with mortality was driving pressure while on ECMO. This suggests that a strategy limiting driving pressure is a reasonable target for clinical practice, but leaves uncertainty as to the role of other strategies and targets such as airway pressure release ventilation (APRV), PEEP titration and monitoring of mechanical power. In the two largest trials of VV ECMO in ARDS, CESAR trial^16^ allowed more variability in MV practices, while the EOLIA trial^17^ implemented a protocolised protective ventilation strategy in the ECMO arm, which prioritised lung rest, but did allow alternatives such as APRV.

**Recommendation 4**

Mechanical ventilation during treatment with VV ECMO should maximise lung protective ventilation. ECMO treatment should be used to facilitate a reduction in parameters associated with lung injury, such as driving pressure, tidal volume and mechanical power. Acknowledging that high driving pressures seen in ARDS may be a marker of disease severity and therefore not necessarily modifiable.

*Good practice statement*.

### 6.3 Expert opinion on clinical application

No recommendation could be made based on the literature reviewed due to the lack of comparator studies generally. Given the priority to issue practical guidance for clinicians using VV ECMO, the GDG considered Dewidar et al. criteria for making a good practice statement.^53^ Evidence from both ARDS patients without ECMO and landmark ECMO trials supports “lung-rest” strategies. This is consistent with existing GRADE-based ARDS guidelines,^54^^,^^55^ and ELSO opinion-based guidance.^56^ Existing literature does not support the development of a de novo MA and would be unlikely to change the strong indirect evidence supporting lung-protective ventilation principles in this setting. In the absence of more recent comparator data between ventilation strategies during ECMO, these observational and trial-based insights support a consistent principle: MV settings should be individualised but minimised to reduce harm. Reasonable strategies include those used in positive trials and those limiting driving pressure. In this context, parameters such as tidal volume, driving pressure and mechanical power should be interpreted as markers of lung stress rather than proven causal targets, particularly during VV ECMO where ventilatory variables can be substantially modified independent of lung recovery.

Notable trials of VV-ECMO in adults with ARDS used the following parameters, which may guide practice:

• CESAR trial^16^ intervention limited Pplat 20-25cmH_2_O, PEEP 10cmH_2_O, respiratory rate of 10/min and F_i_O_2_ 0.3
• EOLIA trial^17^ intervention limited Pplat ≤24cmH_2_O, PEEP ≥10cmH_2_O, respiratory rate of 10–30/min and F_i_O_2_ 0.3–0.5

Subgroup considerations further highlight the need for tailored approaches. A subset of patients treated with VV ECMO may be extubated during extracorporeal support and managed without MV, a strategy that may facilitate early rehabilitation, mobilisation, communication and nutrition, while avoiding the deleterious effects of prolonged invasive ventilation.^57^ Identifying which patients can tolerate ‘awake ECMO’ remains an area of active research (REDEEM *NCT05562505*). Additionally, patients on VV ECMO for non-ARDS causes of hypoxic respiratory failure, such as lung transplantation recipients, may require different ventilatory approaches. However, in the absence of specific evidence, the application of standard lung-protective principles to prevent VILI remains a reasonable and pragmatic approach in these populations.

### 6.4 Unresolved questions and research gaps

There is a lack of high-certainty evidence directly evaluating distinct ventilation strategies in VV ECMO treated patients with ARDS. Most available evidence is observational, highly heterogeneous and focuses on associations rather than comparator trials of strategies. Key unresolved questions include identifying which specific ventilatory parameters (*e.g.*, driving pressures, mechanical power or PEEP) are most critical to minimise VILI in the context of VV ECMO, and what the safe upper limits are for those parameters on ECMO It also remains uncertain whether protocolised ventilation strategies can be universally applied, or if ventilator settings should instead be individualised based on patient, disease or ECMO factors. Furthermore, the extent to which different ventilation strategies impact not only short-term outcomes but also long-term pulmonary and functional outcomes is a priority for further research.

## 7 Conclusions

In conclusion, this first instalment of a three-part guideline series presents evidence-based recommendations and good practice statements for the indications for and management of VV ECMO. Key research gaps have been identified, and future research priorities are outlined for each clinical question to support ongoing improvements in clinical practice and patient outcomes.

## CReDiT authorship contribution statement

Sally F. Newman conceptualisation, investigation, formal analysis, writing – original draft, project administration; Aidan Burrell formal analysis, review and editing; Hergen Buscher conceptualisation, investigation, formal analysis, writing –review and editing; Daniel Thomas Chung conceptualisation, investigation, formal analysis, writing –review and editing; Paul Forrest formal analysis, review and editing; John Fraser conceptualisation, funding acquisition; Craig French investigation, formal analysis, review and editing; Carol Hodgson funding acquisition, formal analysis, review and editing; Ryan Ruiyang Ling formal analysis, investigation, writing – review and editing; Ed Litton formal analysis, review and editing; Graeme MacLaren formal analysis, investigation, writing – review and editing; Andrew McKee formal analysis, review and editing; Zachary Munn methodology, validation; Nhi Nguyen formal analysis, review and editing; Julia K. Pilowski investigation; Kollengode Ramanathan formal analysis, investigation, writing – review and editing; Mark Sackley formal analysis, review and editing; Kiran Shekar formal analysis, review and editing; Myles Smith conceptualisation, investigation, formal analysis, writing – review and editing; Nikki Stamp formal analysis, review and editing; Madeline Wilkinson conceptualisation, investigation, formal analysis, writing –review and editing; Bruce Wilson investigation; Priya Nair conceptualisation, investigation, formal analysis, writing –review and editing, supervision.

## Funding statement

The work was funded by the ACTIONS-CRE at the University of Queensland and CRE-ICU (GNT 1196602) at the ANZIC-RC. The funders had no part in the study design, conduct or data analysis and did not have any authority over these activities.

## Conflict of interest

The authors declare the following financial interests/personal relationships which may be considered as potential competing interests: Carol Hodgson and Ed Litton declare they are part of the CC&R editorial team as associate editors If there are other authors, they declare that they have no known competing financial interests or personal relationships that could have appeared to influence the work reported in this paper. Sally Newman reports that financial support was provided by Monash University, Intensive Care CRE. John Fraser declares research support including grants from Mallinkodt, MERA, Xenios to the Critical Care Research Group, related to the research matter but not directly to this manuscript.
