# Supplementary material for: Evidence-based guidelines for the use of extracorporeal membrane oxygenation in Australia and New Zealand using GRADE methodology series part 1: Venovenous extracorporeal membrane oxygenation (VV ECMO) indications and management
Source: Crit Care Resusc. 2026 Feb 26;28(1):100163. doi: 10.1016/j.ccrj.2026.100163 (PMC12955552; doi:10.1016/j.ccrj.2026.100163)
Supplement: Multimedia component 1 [file mmc1.docx]

**Guidelines for the use of extracorporeal membrane oxygenation in Australia and New Zealand using GRADE methodology**

**Series Part 1: Venovenous extracorporeal membrane oxygenation indications and management**

**Supplementary File**

Introduction

These guidelines for the use of venovenous extracorporeal membrane oxygenation (VV ECMO) are informed by the literature and provide a summary of the most up-to-date evidence available up to August 2024. The methodology used to develop these guidelines is described in detail in a protocol published in Critical Care and Resuscitation,^1^ and describes the processes used to formulate the clinical questions, access the evidence and derive the recommendations.

Management of potential conflicts of interest

In accordance with the National Health and Research Medical Council (NHMRC) standards for developing clinical practice guidelines, all members of the guideline development group (GDG) were required to fully declare any actual, potential, or perceived conflict of interest (COI) (Table 1). The World Health Organisation (WHO) Declaration of Interest for WHO Experts form was used for this purpose.^2^ All completed declarations were reviewed and COIs were discussed explicitly on a case-by-case basis to assess any potential impact on the integrity of the guideline process. For the clinical questions addressed in this series, no members reported relevant COI, and no individuals were recused from voting or participation in decision-making.

| Name | Position/Organisation | Conflicts Declared |
| --- | --- | --- |
| A/Prof Priya Nair | Senior Specialist Intensive Care Services  St Vincent’s Hospital, Sydney  Faculty of Medicine, UNSW, Sydney  The George Institute for Global Health | Publications – Academic research, many publications; Speeches/lectures – multiple conferences. Management committee- EXCEL, BLENDER, other ECMO trials. ACI NSW ECMO advisory group |
| A/Prof Hergen Buscher | Director of Intensive Care  St Vincent’s Hospital, Sydney  Centre of Applied Medical Research, UNSW, Sydney | Publications – Academic research, many publications; Speeches/lectures – multiple conferences |
| A/Prof Aidan Burrell | Senior Specialist Intensive Care  Alfred Hospital, Melbourne  ANZIC-Research Centre | Research support from NHMRC (EXCEL registry, BLENDER trial); Research support Monash Institute of Medical Engineering (ECMO Simulation, Thrombosis in ECMO) |
| Dr Daniel Thomas Chung | Intensive Care Advanced Trainee Royal Prince Alfred Hospital, Sydney  Adjunct Associate Lecturer St Vincent's Clinical School UNSW | None declared |
| A/Prof Paul Forrest | Cardiac Anaesthetist and Perfusionist  Royal Prince Alfred, Sydney | Research support Stryker and Getinge (eCPR materials only); Stryker (economic analysis of eCPR unrestricted); Publications – academic research, many publications; Speeches/lectures – multiple occasions; Development of related guidelines (eCPR); NSW Agency for Clinical Innovation committee – Model of care for ECMO in NSW; NSW ECMO retrieval service |
| Prof John Fraser | Director, Critical Care Research Group, The Prince Charles Hospital & University of Queensland, Australia | Immediate past president Asia Pacific ELSO (APELSO) (unpaid); Research support including grants, collaborations, sponsorships and other funding: Mallinkodt, MERA, Xenios grants to research group, no personal benefit; fund ECMO research |
| A/Prof Craig French | Director of Intensive Care Medicine  Western Health Services, St Albans  Honorary Clinical Professor, Department of Critical Care, University of Melbourne, Parkville VIC | None declared |
| Prof Carol Hodgson | Executive Director, Monash Partners Professor of Intensive Care Research, Head of the Division of Clinical Trials and Cohort Studies Deputy Director ANZIC-RC, Monash University, VIC | Publications – Academic research, many publications; Speeches/lectures – multiple conferences; Expert testimony;  Development of related guidelines; Chair EXCEL Committee; Management committee BLENDER |
| Dr Ryan Ruiyang Ling | Yong Loo Lin School of Medicine, National University of Singapore, Singapore;  Australian and New Zealand Intensive Care Research Centre, School of Public Health and Preventive Medicine, Monash University, Melbourne, VIC Australia | None declared |
| A/Prof Ed Litton | Head of Intensive Care Research and Senior Staff Specialist Intensive Care  Fiona Stanley Hospital, Perth | Publications – Academic research, many publications; EXCEL management committee; |
| A/Prof Graeme MacLaren | Director of Cardiothoracic ICU and ECMO, National University Hospital, Singapore  Adjunct Professor Yong Loo Lin School of Medicine, National University of Singapore, Singapore; | Serves on the Board of Directors of the Extracorporeal Life Support Organisation (unpaid) |
| Dr Andrew McKee | Clinical Director Cardiovascular Intensive Care (Intensivist and Cardiac Anaesthetist)  Auckland City Hospital | None declared |
| Prof Zachary Munn | Director Adelaide GRADE Centre  Director Health Evidence Synthesis,  Recommendations and Impact (HESRI), University of Adelaide | Employment - Adelaide GRADE Centre,  Past-Chair of Guidelines International Network both develop evidence-based guidelines |
| Sally Newman | ECMO Project Manager, St Vincent’s Hospital, Sydney | None declared |
| Dr Nhi Nguyen | Clinical Director of Intensive Care NSW  Agency of Clinical Innovation | Development of practice guides and model of care development at the request of the Ministry of Health NSW |
| Julia Pilowsky | School of Public Health, Faculty of Medicine and Health, University of Sydney, Australia; Intensive Care NSW, NSW Agency for Clinical Innovation, Australia | None declared |
| A/Prof Kollengode Ramanathan | Adjunct Associate Professor, Yong Loo Lin School of Medicine, National University of Singapore, Singapore; Cardiothoracic Intensive Care Unit, National University Heart Centre, National University Health System, Singapore | None declared |
| Prof Kiran Shekar | Intensive Care Specialist  Prince Charles Hospital, Brisbane  Adult Intensive Care Services, School of Medicine, Queensland University of Technology, Gold Coast, Australia; Faculty of Medicine, University of Queensland, Gold Coast, Australia; Faculty of Health Sciences and Medicine, Bond University, Gold Coast, Australia | None declared |
| Dr Myles Smith | Consultant Intensivist  Dunedin Hospital, New Zealand  Clinical Senior Lecturer  Otago University, Otago, New Zealand | Publications – academic research multiple publications |
| Dr Nikki Stamp | Cardiothoracic Surgeon Alfred Hospital, Melbourne  Adjunct Clinical Senior Lecturer, Curtin University | Previous employment with AstraZeneca |
| Mr Mark Sackley | Consumer representative | None declared |
| Dr Bruce Wilson | Critical Care Senior Resident, Tweed Valley Hospital, Cudgen, NSW Australia | None declared |
| Dr Madeline Wilkinson | Cairns and Hinterland Hospital and Health Services District, QLD, Australia | None declared |

Table 1. Composition of GDG and Evidence Team and their disclosed COI

Clinical Questions

The clinical questions addressed in this guideline were developed and prioritised using the Grading of Recommendations Assessment, Development and Evaluation (GRADE) approach within the GRADEpro Guideline Development Tool (GDT). An initial list of candidate questions was generated by members of the GDG using the population, intervention, control and outcome (PICO) structure for binary comparisons or in a free-form manner for open-ended questions. Each question was carefully refined to ensure precise definition of the intended population, intervention, comparator and scope. The GDG then independently rated each question on a 9-point priority scale using GRADEpro GDT (1-3 low, 4-6 moderate, 7-9 high). Median scores >6 were advanced for full guideline development; those with a median 4-6 were retained on a “to-be-considered” list for possible future work and those with a median <4 were excluded. The questions to be covered in this part of the three-series focused on the indications for and management of VV-ECMO in adult patients (Table 2).

| Core question 1: In the management of adult patients with severe hypoxic respiratory failure, should VV ECMO or usual care alone be used? |
| --- |
| Core question 2: In the management of adult patients with severe hypercapnic respiratory failure, should VV ECMO or usual care alone be used? |
| Core question 3: In the management of adult patients with ARDS receiving VV ECMO, should prone positioning or usual care alone be used? |
| Core question 4: In the management of adult patients with ARDS receiving VV ECMO, what is the best mechanical ventilation strategy? |

Table 2. Core questions covered in Part 1 of series

Outcomes

During the first stage of this project, the GDG was tasked with generating and brainstorming outcomes for each clinical question. The group was united in its decision to include core outcome measures suggested in an international, modified Delphi study, as illustrated in Table 3.^3^ The core outcome measures fell into three domains: death, adverse effects and life impacts.

There is inconsistency in outcome reporting, with studies using varying definitions for critical endpoints such as functional recovery and long-term quality of life. This heterogeneity limits the ability to accurately assess the benefits of ECMO, particularly in understanding its long-term effects.

We intentionally kept the term "functional outcome" broad to allow for a comprehensive and inclusive assessment of the literature. This approach ensured that we could capture a wide range of relevant findings, rather than limit the analysis to a single predefined measure. We aimed to better reflect the diversity of patient experiences and provide a more comprehensive evidence base for informed decision-making. However, when the outcome measures differed, the ability to compare or combine results became difficult or impossible, undermining the evidence base for the recommendation.

Once the evidence was gathered, the GDG reassessed the relative importance of outcomes initially rated as critical to decision-making. Based on feedback from the panel, placing significant weight on our consumer representatives, some outcomes were downgraded from critical to important but were still included in the guideline, and these deliberations informed the final strength and direction of recommendations where initial voting was not unanimous.

| Core question | Desired critical-rated outcomes | Actual reported outcomes |
| --- | --- | --- |
| In the management of adult patients with severe hypoxic respiratory failure, should VV ECMO or usual care alone be used? | Short-term mortality (≤90 days)  Long-term mortality (>90 days)  Functional Outcome (Downgraded- Important)  Quality of Life  Neurological Injury  Hospital length of stay (Downgraded- Important)  Cost (Downgraded- Important) | 28-day mortality  90-day mortality  Neurological Injury  Quality of Life SF-36  Long-term mortality (1 year)  Cost per QALY  Hospital length of stay  Functional outcome |
| In the management of adult patients with severe hypercapnic respiratory failure, should VV ECMO or usual care alone be used? | Long-term mortality (>90 days)  Short-term mortality (≤90 days)  Functional Outcome  Ability to apply lung-protective ventilation  Quality of Life  Neurological Injury  Bleeding complications  Mechanical Ventilation Duration  Hospital length of stay |  |
| In the management of adult patients with ARDS receiving VV ECMO, should prone positioning or usual care alone be used? | Short-term mortality (≤90 days)  Long-term mortality (>90 days)  Functional Outcome  Mechanical Ventilation Duration  Proning-related complications  ECMO duration  Quality of Life | Mortality (at longest follow-up)  Long-term mortality (>90 days)  ECMO duration |

^QALY: quality life adjusted years^

Table 3. Summary of critical outcomes prioritised for each PICO

Core question 4: The GDG recognised that the intervention was not a single therapy but a range of alternative MV strategies during VV ECMO. GRADEpro does not currently support ranking of outcomes for multi-comparison questions. Therefore, no formal rating or prioritisation of outcomes by the GDG was undertaken. Instead, the literature search was structured to capture studies that reported outcomes considered important in ARDS and ECMO research. By ensuring capture of studies reporting mortality, we could systematically identify and extract additional relevant outcomes.

Literature search, data extraction and quality of evidence evaluation

Evidence profiling involved a systematic and hierarchical approach consisting of three phases, with comprehensive literature searches conducted for each phase, as recommended by the NHMRC ‘Guideline for Guidelines’ document.^4^ This process is explained in detail within our protocol.^1^ Publications were selected according to the preselected inclusion criteria for each PICO. Literature screening was undertaken independently by two members of the evidence team. Initially, for the hypoxic respiratory failure PICO, this was performed using EndNote™,^5^ and then the transition was made to Covidence software.^6^ Data from included studies were extracted to Review Manager 5.4 (RevMan)^7^ or R software (version 4.4.2).^8^

The forest plots presented in this guideline incorporate both random and fixed effect models. This variation reflects the approach used in the original systematic reviews and meta-analyses from which the data were adopted.^9^ The same analytical model was retained to ensure consistency with the source analyses. This group performed sensitivity analyses to confirm that the choice of model did not materially alter the direction or magnitude of the pooled estimates. The GDG agreed that formal numerical decision thresholds would not be specified for critical outcomes. Instead, judgements regarding the clinical importance of observed effects were made through consensus, considering the context of ECMO use and the potential for patient relevant benefit or harm. In general, the GDG considered whether the confidence interval crossed the null effect when determining imprecision and the likelihood of a clinically meaningful effect.^10^

Voting on the final wording of the recommendations was done anonymously via an online survey, and given the limited (small) size of the panel, the project management team pre-specified a 100% agreement for all recommendations to limit individuals' influence on the panel. If the vote ≤99%, then a meeting was held to discuss concerns before the members of the GDG underwent a revote.

Following protocol development, the planned use of the ROBINS-I^11^ tool for assessing non-randomised studies was amended to the Newcastle-Ottawa Scale (NOS)^12^ due to the complexity and resource demands of the ROBINS-I assessment. NOS was used as a pragmatic screening tool, while key sources of bias, including confounding and time-dependant bias, were explicitly considered during GRADE certainty assessments.

References

1. Newman SF, Munn Z, French C, Buscher H, Chung DT, Smith M, et al. Protocol for the development of NHMRC-endorsed guidelines for extracorporeal membrane oxygenation using GRADE methodology. Crit Care Resusc. 2025 Mar 1;27(1):100093.

2. Declaration of interests for WHO experts [Internet]. [cited 2025 Feb 6]. Available from: https://www.who.int/publications/m/item/declaration-of-interests-for-who-experts

3. Hodgson CL, Burrell AJC, Engeler DM, Pellegrino VA, Brodie D, Fan E, et al. Core Outcome Measures for Research in Critically Ill Patients Receiving Extracorporeal Membrane Oxygenation for Acute Respiratory or Cardiac Failure: An International, Multidisciplinary, Modified Delphi Consensus Study. Crit Care Med. 2019 Nov;47(11):1557–63.

4. Guidelines for Guidelines | NHMRC [Internet]. [cited 2023 Feb 17]. Available from: https://www.nhmrc.gov.au/guidelinesforguidelines

5. The EndNote Team EndNote. EndNote X9 ed. [Internet]. EndNote X9. ed. Philadelphia, PA: Clarivate. 2013. Available from: https://support.clarivate.com/Endnote/s/article/Download-EndNote?language=en_US

6. Covidence - Better systematic review management [Internet]. Covidence. [cited 2023 Nov 28]. Available from: https://www.covidence.org/

7. The Cochrane Collaboration, 2020. Review Manager (RevMan).

8. R: The R Project for Statistical Computing [Internet]. [cited 2025 Oct 23]. Available from: https://www.r-project.org/

9. Schünemann HJ, Wiercioch W, Brozek J, Etxeandia-Ikobaltzeta I, Mustafa RA, Manja V, et al. GRADE Evidence to Decision (EtD) frameworks for adoption, adaptation, and de novo development of trustworthy recommendations: GRADE-ADOLOPMENT. J Clin Epidemiol. 2017 Jan;81:101–10.

10. Guyatt GH, Oxman AD, Kunz R, Brozek J, Alonso-Coello P, Rind D, et al. GRADE guidelines 6. Rating the quality of evidence--imprecision. J Clin Epidemiol. 2011 Dec;64(12):1283–93.

11. Sterne JAC, Hernán MA, Reeves BC, Savović J, Berkman ND, Viswanathan M, Henry D, Altman DG, Ansari MT, Boutron I, Carpenter JR, Chan AW, Churchill R, Deeks JJ, Hróbjartsson A, Kirkham J, Jüni P, Loke YK, Pigott TD, Ramsay CR, Regidor D, Rothstein HR, Sandhu L, Santaguida PL, Schünemann HJ, Shea B, Shrier I, Tugwell P, Turner L, Valentine JC, Waddington H, Waters E, Wells GA, Whiting PF, Higgins JPT. ROBINS-I: a tool for assessing risk of bias in non-randomized studies of interventions. Br Med J. 2016;355(4919).

12. Ottawa Hospital Research Institute [Internet]. [cited 2025 Aug 25]. Available from: https://www.ohri.ca/programs/clinical_epidemiology/oxford.asp

**Core question 1**

**In the management of adult patients with severe hypoxic respiratory failure, should VV ECMO or usual care alone be used?**

Literature search and evaluation

Phase 1 search for existing guidelines, nine guideline portals (GIN library,^1^ ECRI Guidelines Trust,^2^ Database of GRADE EtD’s and Guidelines,^3^ MAGICapp,^4^ BIGG International database of GRADE guidelines,^5^ NICE guidelines,^6^ NHMRC Guidelines portal (decommissioned), ELSO Guidelines,^7^ and TRIP database^8^ were searched using the search string “extracorporeal membrane oxygenation” and “ECMO”. The inclusion period was from inception up to May 2021. A total of four guidelines were identified.

**Identification of studies via databases and registers**

Records removed *before screening*:

Duplicate records removed (n = 55)

Records identified from:

Databases (n = 365)

**Identification**

Records screened

(n = 310)

Records excluded

(n = 164)

Reports sought for retrieval

(n = 146)

Reports excluded: (n= 119)

Not addressing ECMO,

Paediatric, not guideline or no recommendation, not based on SR

**Screening**

Reports assessed for eligibility

(n = 27)

Reports excluded (n=26) not addressing core question or outcomes specified

Studies included in review

(n = 4)

**Included**

Figure 1. PRISMA flow chart of guidelines for core question 1

| **Title** | **Author** | **Year** | **Use of GRADE** | **Selected outcomes addressed** |
| --- | --- | --- | --- | --- |
| Extracorporeal membrane oxygenation for severe acute respiratory failure in adults^9^ | NICE | 2011 | No | Mortality |
| Guidelines on the management of acute respiratory distress syndrome^10^ | Faculty of Intensive Care Medicine / Intensive Care Society | 2018 | Yes | Mortality |
| An Official American Thoracic Society/European Society of Intensive Care Medicine/Society of Critical Care Medicine Clinical Practice Guideline: Mechanical Ventilation in Adult Patients with Acute Respiratory Distress Syndrome^11^ | American Thoracic Society/European Society of Intensive Care Medicine/Society of Critical Care Medicine | 2017 | Yes | Mortality |
| Mechanical Ventilation and Extracorporeal  Membrane Oxygenation in Acute Respiratory  Insufficiency^12^ | Association of the Scientific Medical Societies in Germany | 2018 | Yes | Mortality |

Table 1. Summary of guidelines considered for core question 1

These four guidelines addressed the use of VV ECMO in severe hypoxic respiratory failure with mortality as the sole reported outcome. Publication of these guidelines preceded the release of one or both of the two significant randomised controlled trials (RCTs)^13,14^ completed within this domain and therefore deemed to lack the depth of current available evidence and not suitable for GRADE ADOLOPMENT.^15^

Phase 2 search for existing systematic reviews (SR) for core question 1 was completed using two Systematic Review Databases, Epistemonikos^16^ and PROSPERO^17^ using the search string “extracorporeal membrane oxygenation” and “ECMO”. The inclusion period was from inception to March 2021.


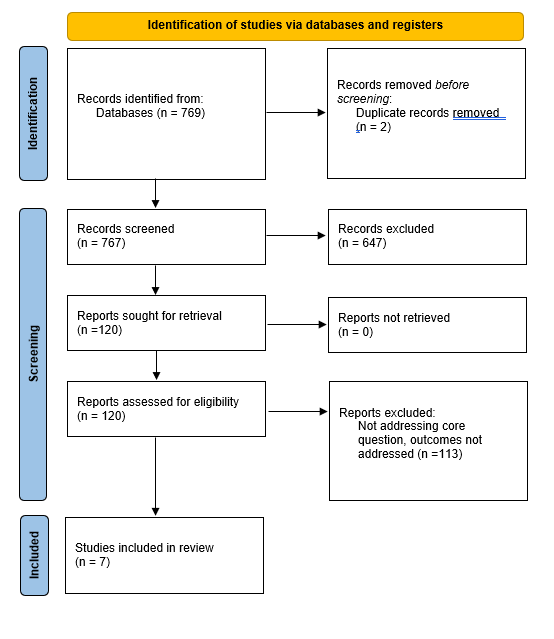


Figure 2. PRISMA flow chart of SRs for core question 1

There were seven SRs that addressed the use of VV ECMO in severe hypoxic respiratory failure. The Joanna Briggs Institute (JBI) Critical Appraisal checklist for SR^18^ and research synthesis was used to determine suitability. All were found acceptable for inclusion.

| **Title** | **Author** | **Year** | **Outcomes addressed** |
| --- | --- | --- | --- |
| ECMO for severe ARDS: systematic review and individual patient data meta-analysis^19^ | Combes et al. | 2020 | 90-day mortality  28-day mortality  60-day mortality  Hospital length of stay |
| Extracorporeal membrane oxygenation for severe acute respiratory distress syndrome in adult patients: a systematic review and meta-analysis^20^ | Mendes et al. | 2019 | Mortality (latest reported)  Hospital length of stay |
| Venovenous extracorporeal membrane oxygenation for acute respiratory distress syndrome: a systematic review and meta-analysis^21^ | Munshi et al. | 2019 | 30-day mortality  60-day mortality  Mortality (latest follow-up)  Neurological injury |
| Systematic review and meta-analysis of complications and mortality of venovenous extracorporeal membrane oxygenation for refractory acute respiratory distress syndrome^22^ | Vaquer et al. | 2017 | Hospital mortality  Neurological injury  (Intracranial haemorrhage) |
| Is Extracorporeal Membrane Oxygenation the Standard Care for Acute Respiratory Distress Syndrome: A Systematic Review and Meta-Analysis^23^ | Wang et al. | 2021 | 60-day mortality  1 year mortality  ICU mortality  Hospital mortality  Neurological injury  (haemorrhagic stroke) |
| Long-Term Quality of Life After Extracorporeal Membrane Oxygenation in ARDS Survivors: Systematic Review and Meta-Analysis^24^ | Wilcox et al. | 2020 | Quality of life (SF-36, PCS, MCS)  Functional outcome (FVC, FEV1, anxiety, depression) |
| Extracorporeal membrane oxygenation versus mechanical ventilation alone in adults with severe acute respiratory distress syndrome: a systematic review and meta-analysis^25^ | Zhu et al. | 2021 | 90-day mortality  30-day mortality  Hospital mortality |

^ARDS: Acute Respiratory Distress Syndrome; SF-36: 36-Item Short Form Survey; PCS: Physical Component Summary; MCS: Mental Component Summary; FVC: Forced Vital Capacity; FEV1: Forced Expiratory Volume in one sec;^

Table 2. Summary of SRs considered for core question 1

| JBI Checklist | Combes 2020 | Mendes 2019 | Munshi 2019 | Vaquer 2017 | Wang 2020 | Wilcox 2020 | Zhu 2021 |
| --- | --- | --- | --- | --- | --- | --- | --- |
| 1. Is the review question clearly and explicitly stated? | 🟢 | 🟢 | 🟢 | 🟢 | 🟢 | 🟢 | 🟢 |
| 2. Were the inclusion criteria appropriate for the review question? | 🟢 | 🟢 | 🟢 | 🟢 | 🟢 | 🟢 | 🟢 |
| 3. Was the search strategy appropriate? | 🟢 | 🟢 | 🟢 | 🟢 | 🟢 | 🟢 | 🟢 |
| 4. Were the sources and resources used to search for studies adequate? | 🟢 | 🟢 | 🟢 | 🟢 | 🟢 | 🟢 | 🟢 |
| 5. Were the criteria for appraising studies appropriate? | 🟢 | 🟢 | 🟢 | 🟢 | 🟢 | 🟢 | 🟢 |
| 6. Was critical appraisal conducted by two or more reviewers independently? | 🟢 | 🟢 | 🔴 | 🟢 | 🔴 | 🟢 | 🟢 |
| 7. Were there methods to minimize errors in data extraction? | 🟢 | 🟢 | 🟢 | 🟢 | 🟢 | 🟢 | 🟢 |
| 8. Were the methods used to combine studies appropriate? | 🟢 | 🟢 | 🟢 | 🟢 | 🟢 | 🟢 | 🟢 |
| 9. Was the likelihood of publication bias assessed? | 🔴 | 🔴 | 🟢 | 🟢 | 🔴 | 🟢 | 🟢 |
| 10. Were recommendations for policy and/or practice supported by the reported data? | 🟢 | 🟢 | 🟢 | 🟢 | 🟢 | 🟢 | 🟢 |
| 11. Were the specific directives for new research appropriate? | 🟢 | 🔴 | 🔴 | 🟢 | 🟢 | 🟢 | 🟢 |
| Overall appraisal | 10/11 (include) | 9/11 (include) | 9/11  (include) | 11/11  (include) | 10/11 (Include) | 11/11 (Include) | 11/11  (include) |

^🟢 = yes 🔴 = no 🟡 = unclear; overall score ≥9 included in review^

Table 3. JBI Critical Appraisal checklist for SRs reviewed in core question 1

Authors Peek and Combes combined individual data from the two RCTs^13,14^ to perform a meta-analysis (MA), which was selected to provide evidence for 28-day mortality, 90-day mortality and hospital length of stay.^19^ Data on one-year mortality came from Wang et al.,^23^ which also provided evidence on haemorrhagic stroke that informed the outcome of neurological injury. Quality of life was thoroughly addressed by Wilcox et al.^24^ through the assessment of the SF-36 instrument components. Wilcox et al.^24^ also provided evidence for functional outcome variables using validated instruments to determine lung function (FEV1, FVC) as well as anxiety and depression (measured with the Hospital Anxiety and Depression scale (HADs)).

As cost was not addressed in any SR, a phase 3 search for primary literature was completed. This phase was completed by systematically searching three databases, Medline,^26^ Embase,^27^ and CENTRAL^28^ from 1996 to October 2020. A relevant study was found and later added in February 2022.

Phase three Medline,^26^ Embase^27^ and CENTRAL^28^ were searched with the following search strings:

Medline

1. Extracorporeal membrane oxygenation/
2. Ecmo.mp.
3. Extracorporeal membrane oxygenation.mp.
4. Extracorporeal oxygenation.mp.
5. 2 or 3 or 4
6. Limit 5 to yr=”2018-current”
7. 1 or 6
8. limit 7 to “all adult (19 plus years)”
9. limit 8 to English language
10. hypoxia/
11. hypoxic respiratory failure.mp.
12. hypoxia.mp.
13. 11 or 12
14. Limit 13 to yr=”2018-current”
15. 10 or 14
16. Limit 15 to English language
17. 16 and 9

Embase

1. Extracorporeal oxygenation/
2. Ecmo.mp.
3. Extracorporeal membrane oxygenation.mp.
4. Extracorporeal oxygenation.mp.
5. 2 or 3 or 4
6. Limit 5 to yr=”2018-current”
7. 1 or 6
8. Limit 7 to conference abstracts
9. 7 not 8
10. Limit 9 to medline
11. 9 not 10
12. Limit 11 to (article or article in press or ‘review’)
13. Limit 12 to English language
14. Limit 13 to (adult <18 to 64 years> or aged <65+ years>)
15. Hypoxia/
16. Hypoxic respiratory failure.mp.
17. Hypoxia.mp.
18. 16 or 17
19. Limit 18 to yr=”2018-current”
20. 15 or 19
21. Limit 20 to conference abstracts
22. 20 not 21
23. Limit 22 to medline
24. 22 not 23
25. Limit 24 to (article or article in press or “review”)
26. Limit 25 to English language
27. Limit 26 to (adult <18 to 64 years> or aged <65+ years)
28. 14 and 27

CENTRAL

1. Extracorporeal membrane oxygenation/
2. Extracorporeal oxygenation.mp.
3. Ecmo.mp.
4. Extracorporeal membrane oxygenation.mp.
5. 2 or 3 or 4
6. Limit 5 to yr=”2018- current” [limit not valid in DARE; records were retained]
7. 1 or 6
8. Limit 7 to (“adult (19 to 44 years)” or “middle age (45 to 64 years)” or middle aged (45 plus years)” or “all aged (65 and over)”) [limit not valid in CDSR, ACP Journal Club, DARE, CCA, CCTR, CLCMR; records were retained]
9. Limit 8 to English language [limit not valid in CDSR, ACP Journal Club, DARE, CCA, CCTR, CLCMR; records were retained]
10. Hypoxia/
11. Hypoxic respiratory failure.mp.
12. Hypoxia.mp.
13. 11 or 12
14. Limit 13 to yr=”2018- current” [limit not valid in DARE; records were retained]
15. 10 or 14
16. Limit 15 to (“adult (19 to 44 years)” or “middle age (45 to 64 years)” or middle aged (45 plus years)” or “all aged (65 and over)”) [limit not valid in CDSR, ACP Journal Club, DARE, CCA, CCTR, CLCMR; records were retained]
17. Limit 16 to English language [limit not valid in CDSR, ACP Journal Club, DARE, CCA, CCTR, CLCMR; records were retained]
18. 9 and 17


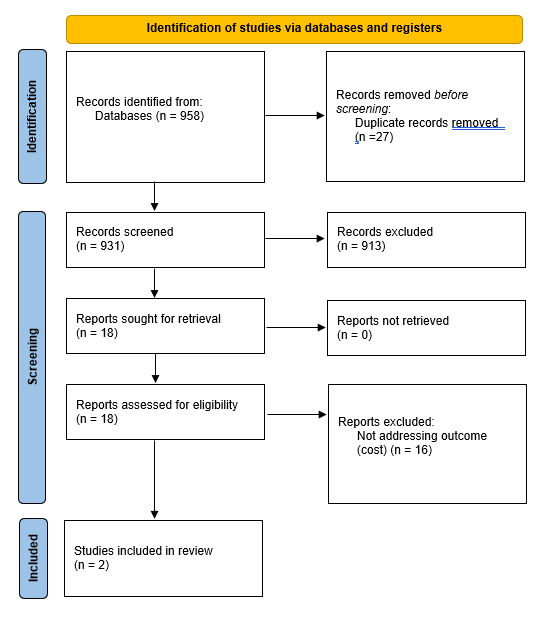


Figure 3. PRISMA flow chart of primary literature for core question 1

Two studies examined the cost of VV ECMO compared to usual care alone in severe hypoxic respiratory failure. One was an observational study,^29^ and the other was the CESAR RCT^30^ with parallel economic evaluation, which was selected as the most robust evidence for this outcome. The risk of bias was considered low when assessed using the Cochrane Risk of Bias version 2.0 (RoB 2.0).^31^ The study profiles are shown below.

| **Title** | **Author** | **Year** | **Outcomes addressed** |
| --- | --- | --- | --- |
| Efficacy and economic assessment of conventional ventilatory support versus extracorporeal membrane oxygenation for severe adult respiratory failure (CESAR): a multicentre randomised controlled trial^29^ | Peek et al. | 2009 | Cost |

Table 4. Summary of primary studies reviewed for core question 1

| **Study** | **D1** | **D2** | **D3** | **D4** | **D5** | **Overall** |
| --- | --- | --- | --- | --- | --- | --- |
| **Peek et al., 2009** | 🟢 | 🟢 | 🟢 | 🟢 | 🟢 | 🟢 |

^D1: bias arising from the randomisation from randomisation process^

^D2: bias due to deviations from intended intervention^

^D3: bias due to missing outcome data^

^D4: bias in measurement of the outcome^

^D5: bias in selection of the reported result^

Table 5. RoB 2.0 assessment of RCT for core question 1

Synthesis of evidence

For numerical results, pooled effect sizes and 95% confidence intervals (CI) were presented where possible. Relative risk (RR) was applied for dichotomous outcomes, while mean differences (MD) were applied to continuous outcomes measured on the same scale. For studies that did not report mean and standard deviation (SD) directly, these were estimated from summary data using the method described by Wan et al., prior to meta-analysis.^32^ Heterogeneity among pooled results was assessed using the I² statistic. In cases where pooling was not possible, heterogeneity was evaluated by examining the size and direction of effects across individual studies. If no high-quality comparative studies were available, cohort studies and case series were included to provide descriptive data on the prognosis of the intervention in relevant subgroups.

Following evidence synthesis, the data were presented to the GDG, where it was decided to redefine the mortality endpoints so the recommendation would rely more heavily on the most robust evidence available, Combes et al.,^19^ including the individual patient data from the two RCTs, rather than systematic reviews based on observational data. The updated mortality endpoints were 28-day mortality, 90-day mortality and long-term mortality measured at one year.

Further discussions took place regarding the relative importance of outcomes. The GRADE process indicates that all outcomes rated as critical to decision-making should be considered when making a recommendation. Initially, the GDG selected seven outcomes as critical for decision-making concerning the use of VV ECMO in severe hypoxic respiratory failure. These were short-term mortality, long-term mortality, functional outcome, quality of life, neurological injury, hospital length of stay, and cost. With significant weight given to the voices of consumers on the panel, the group downgraded the following outcomes: functional outcome, hospital length of stay, and cost. While the panel acknowledged these outcomes as valid, they would not heavily influence the decision to use ECMO in a clinical setting. The panel agreed that these outcomes should still be included in the GRADE evidence profiling.

The use of VV ECMO for severe hypoxic respiratory failure shows a short-term survival benefit, with a reduction in mortality at 28 days (RR 0.57 [95% CI 0.43 to 0.76]) and 90 days (RR 0.78 [95% CI 0.61 to 1.01]). Other critical outcomes addressed, including neurological injury (absolute risk difference -3.9 [95% CI 10.0 to 13]) and quality of life measured by SF-36 (MD 5.40 [95% CI 4.11 to 6.68]) also showed potentially favourable effects of VV ECMO in addition to conventional care in severe hypoxic respiratory failure.


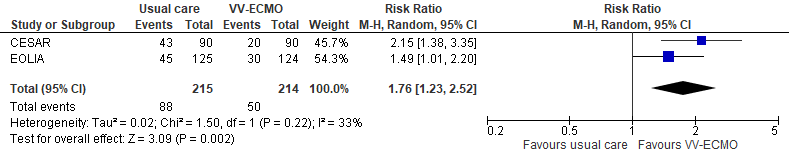


Figure 4. Forest plot outcome: 28-day Mortality


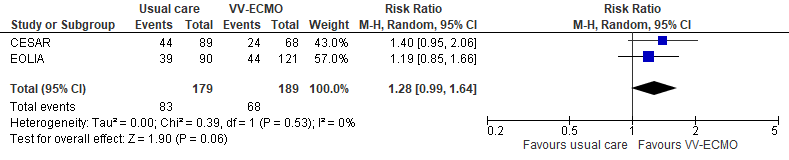


Figure 5. Forest plot outcome: 90-day Mortality


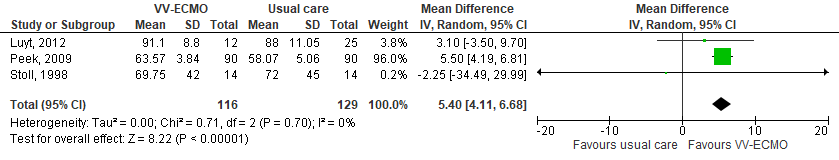


Figure 6. Forest plot outcome: Health Related Quality of Life (HRQoL) (measure by SF-36)


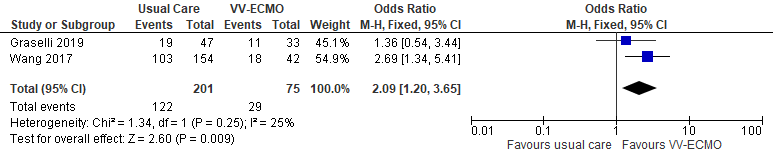


Figure 7. Forest plot outcome: Long-term mortality (1 Year)


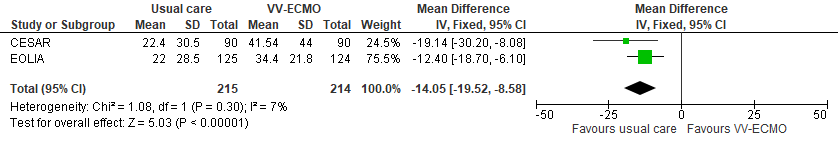


Figure 8. Forest plot outcome: Hospital length of stay


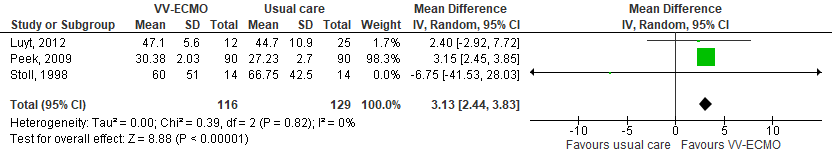


Figure 9. Forest plot outcome: HRQoL (measured by SF-26 PCS)


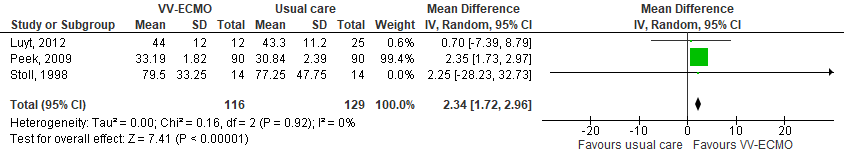


Figure 10. Forest plot outcome: HRQoL (measured by SF-26 MCS)


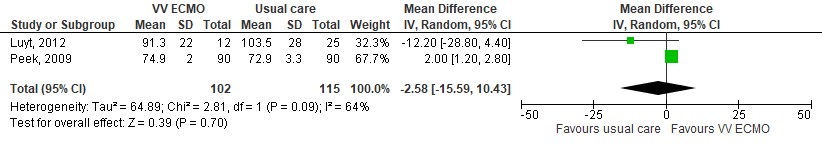


Figure 11. Forest plot outcome: Functional outcome (measured FEV1 % of predicted)


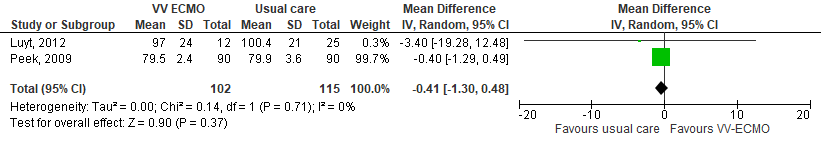


Figure 12. Forest plot outcome: Functional outcome (measured FVC % of predicted)


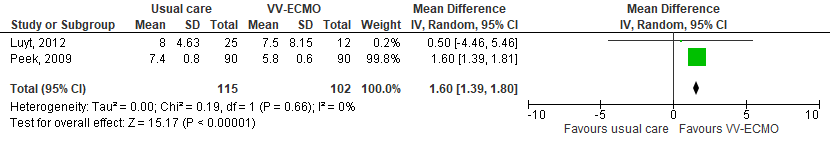


Figure 13. Forest plot outcome: Functional outcome depression (measured with HADs)


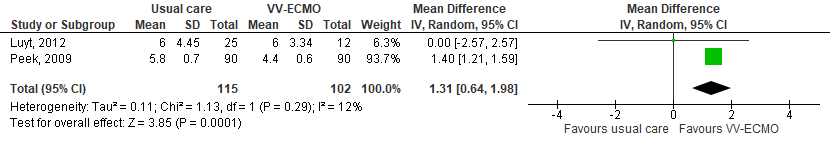


Figure 14. Forest plot outcome: Functional outcomes anxiety (measured with HADs)

Certainty of Evidence

The critical outcomes are shown in the GRADE Summary of Findings (SoF) table below.

In the GRADE framework, imprecision refers to the uncertainty in an effect estimate due to small sample sizes or wide confidence intervals crossing the line of no effect. One key concept in assessing imprecision is the optimal information size (OIS), which refers to the minimum number of participants required to detect a clinically meaningful effect with adequate statistical power. The SRs informing the mortality outcome^19,23^ were downgraded for imprecision due to insufficient sample size. Specifically, the study by Combes et al.^19^ (429 participants) did not meet the calculated OIS threshold of 528, while Wang et al.^23^ included 276 participants, just below the required 280.

This shortfall in participant numbers limits confidence in the precision of the observed effect estimates. In evaluating the outcome of neurological injury in the SR by Wang et al.,^23^ we initially had concerns about the introduction of heterogeneity and potential deviations from the intended intervention, specifically, variations in ECMO management practices across different centres and patient populations. However, we found that the included studies employed appropriate analytical methods, rigorous inclusion criteria and the use of validated tools (SF-36) to address these concerns and mitigate the impact of such variability. Therefore, we decided not to downgrade the certainty of evidence for this outcome. Only one study was available for this outcome, so inconsistency could not be assessed. Although the study reported both appreciable benefits and harm, with wide confidence intervals (CI 95% -10.0 to 1.3) that crossed the threshold of clinical decision-making, we rated down for imprecision due to the uncertainty in the direction and magnitude of the effect.

We used the Peek et al. (CESAR)^30^ study to assess the cost-effectiveness of VV-ECMO for adult patients with severe hypoxic respiratory failure; however, several limitations influenced the low certainty of evidence rating. Imprecision could not be assessed due to the single study. The CESAR trial was conducted in the UK National Health Service, which differs in structure, resource allocation and costs from the Australian healthcare system. This raises concerns about indirectness, understanding that these results may not be fully generalisable to Australia. Although the cost-effectiveness estimate from this study (AUD $35,258 cost per Quality Adjusted Life Year) lies within an acceptable range,^33^ the wide confidence intervals ((95% CI $12,956 - $108,401) GBP from 2009 conversion to AUD November 2021 rate 1.83) reflect a degree of imprecision, which reduced our certainty, and downgraded the GRADE certainty to low.

Wilcox et al.^24^ evaluated long-term HRQoL in survivors of ARDS treated with ECMO using the SF-36 questionnaire. They found that in three studies including 245 patients, ECMO survivors showed fewer impairments in physical health domains, with higher SF-36 scores compared to those who underwent conventional mechanical ventilation alone (MD 5.4 [CI 95% 4.11 to 6.68]). ECMO patients also scored higher in the mental health domains of the SF-36, reporting less depression and anxiety than mechanically ventilated alone patients (MD 2.34 [CI 95% 1.72 to 2.96]), suggesting better HRQoL among ECMO survivors.

During our assessment of Wilcox et al. (2020) systematic review on the long-term quality of life in ECMO survivors,^24^ we identified a discrepancy between the forest plots and the study’s written conclusions. Specifically, the plots indicated that ECMO patients had higher SF-36 scores, indicating better quality of life, yet the text described “greater decrements” in the ECMO group. We contacted the study authors, who confirmed that the forest plots were mislabelled, resulting in incorrect conclusions. Similarly, we identified that the direction of effect in the spirometry outcomes (FEV1 and FVC) was also mislabelled. To verify the findings, we cross-checked the data from the primary sources and re-ran them in RevMan, confirming that ECMO patients consistently demonstrated better outcomes across both physical and mental health domains. Within the spirometry outcomes these outcomes were not statistically significant and did not alter the overall conclusions. Given the accuracy of the underlying data and the limited evidence available in the area, we chose to include the study in our review, clearly documenting the issue and accommodating the potential risk of misinterpretation when applying the GRADE criteria.

Evidence to Decision Framework

To enable the GDG to make a judgment about the direction and strength of the recommendation, the evidence mentioned above is considered in the Evidence to Decision (EtD) framework of the GRADE process.^34,35^ The certainty of evidence supporting the use of VV ECMO in adults with severe hypoxic respiratory failure was judged to be overall very low. This reflects the limited and often varied data available across a wide range of critical outcomes, such as neurological injury and HRQoL. These outcomes are primarily informed by small observational studies, often with methodological limitations that introduce risk of bias and imprecision. However, there was moderate certainty for a survival benefit, based on evidence from two RCTs, which is supported by consistent findings from large international registry studies.

Regarding patient values and preferences, there is no systematically collected evidence. Given the input of consumer representatives on the GDG, the group considered it likely that most well-informed patients facing severe hypoxic respiratory failure and a high risk of death would opt to receive ECMO. Nevertheless, there may be variability in preferences, particularly in patients with substantial comorbidities or frailty. In such cases, the potential for serious harm may lead some patients or families to conclude that the risks outweigh the benefits. The GDG advises that patient preferences be explicitly discussed where possible before ECMO is initiated, and reassessed throughout the treatment. As patients are unlikely to be able to participate in the decision-making process, their substitute decision-makers should be included.

When weighing the balance of benefits and harms, the panel concluded that the use of ECMO probably favours benefit, especially when provided to carefully selected patients at centres with established expertise. ECMO is associated with potentially serious complications, including bleeding, infection, and stroke. Despite these risks, the evidence points to a survival benefit, significant in a life-threatening condition like severe hypoxic respiratory failure. Consequently, the GDG believes that the benefits outweigh the harms for most eligible patients, particularly when administered by experienced multidisciplinary teams following appropriate protocols.

The costs and resource usage associated with ECMO are substantial. Australian data from the H1N1 pandemic estimated an average additional cost of AUD 13,646 per patient for ECMO provision, with total costs reaching AUD 64,207 per patient.^36^ A recent systematic review by Oude Lansink-Hartgring et al.,^37^ assessed the costs associated with ECMO across fourteen studies, four of which specifically examined ECMO for hypoxic respiratory failure. These four studies, originating from the United States, Taiwan, the Netherlands, and the United Kingdom, report a mean cost of approximately USD 101,043 for VV ECMO. Across the included studies, the length of stay in the ICU emerged as the most significant contributor to overall ICU costs, followed by the use of mechanical ventilation. Increased ICU mortality was also linked to substantially higher costs, likely due to the intensive resource usage commonly observed at the end of life. The cost of ECMO-specific equipment accounted for 11% to 20% of the total, while personnel costs were identified as the primary cost driver in two studies.

Equity considerations are important in the context of ECMO. Due to the specialised nature of ECMO, services are typically concentrated in urban tertiary hospitals. This creates potential inequities in access, particularly for rural and regional populations. While some states have developed ECMO retrieval services, others rely on ad hoc solutions. The PROGRESS-PLUS framework^38^ highlights “place of residence” as a likely axis of inequity. Furthermore, access to ECMO also depends on infrastructure, staff training and institutional experience, which can vary significantly across the country. The number of ECMO-capable centres in Australia and New Zealand has increased since the COVID-19 pandemic, potentially improving equitable access.

ECMO is regarded as an acceptable intervention by patients, clinicians and healthcare systems, primarily due to its potential to improve survival in critically ill patients. Nevertheless, given its substantial demands on healthcare resources, ECMO should be implemented exclusively in centres equipped with the necessary infrastructure, specialised clinical expertise and a demonstrated adherence to evidence-based protocols. The feasibility of delivering ECMO is optimised when it is incorporated within a structured and coordinated network, supported by dedicated retrieval systems, allowing timely access for patients outside major centres.

Finally, the GDG underscores the importance of prioritising patient-centred outcomes in future research, with particular emphasis on HRQoL and long-term functional status, areas that remain significantly underreported. Although HRQoL was identified as a critical outcome in this guideline, the certainty of evidence supporting this outcome was low. Importantly, no data reviewed showed that ECMO recipients were disadvantaged in terms of HRQoL outcomes.

|  | **Judgement** | | | | | | |
| --- | --- | --- | --- | --- | --- | --- | --- |
| **Problem** | No | Probably no | Probably yes | **Yes** |  | Varies | Don't know |
| **Desirable Effects** | Trivial | Small | **Moderate** | Large |  | Varies | Don't know |
| **Undesirable Effects** | Large | Moderate | **Small** | Trivial |  | Varies | Don't know |
| **Certainty of evidence** | **Very low** | Low | Moderate | High |  |  | No included studies |
| **Values** | Important uncertainty or variability | Possibly important uncertainty or variability | **Probably no important uncertainty or variability** | No important uncertainty or variability |  |  |  |
| **Balance of effects** | Favors the comparison | Probably favors the comparison | Does not favor either the intervention or the comparison | **Probably favors the intervention** | Favors the intervention | Varies | Don't know |
| **Resources required** | Large costs | **Moderate costs** | Negligible costs and savings | Moderate savings | Large savings | Varies | Don't know |
| **Certainty of evidence of required resources** | Very low | **Low** | Moderate | High |  |  | No included studies |
| **Cost effectiveness** | Favors the comparison | Probably favors the comparison | Does not favor either the intervention or the comparison | **Probably favors the intervention** | Favors the intervention | Varies | No included studies |
| **Equity** | Reduced | Probably reduced | Probably no impact | Probably increased | Increased | **Varies** | Don't know |
| **Acceptability** | No | Probably no | Probably yes | **Yes** |  | Varies | Don't know |
| **Feasibility** | No | Probably no | Probably yes | **Yes** |  | Varies | Don't know |

Table 6. EtD Table of Judgement for core question 1

Subgroup Considerations

Given that severe hypoxic respiratory failure may result from a wide range of underlying pathologies, there may exist significant variation in response to VV ECMO therapy in different subgroups. Available evidence on subgroups is of a lesser quantity and quality than for aggregated patients with severe hypoxic respiratory failure.

*Subgroups considered in RCTs*

Subgroup analyses were conducted in the individual patient data meta-analysis of two major RCTs.^19^ These analyses examined potential effect modifiers, including sex, age (≥49 vs <49 years), primary diagnosis of pneumonia, presence of multiorgan failure (defined as failure of more than 2 organ systems), timing of ECMO initiation (after more than three days of mechanical ventilation), and various markers of disease severity such as PF ratio, lung compliance, PEEP, pH, Murray lung injury score and predicted mortality.

Of the subgroups evaluated, only the presence of multiorgan failure demonstrated a significant interaction with treatment effect. Specifically, patients with greater than two organ failures at the time of randomisation did not appear to benefit from VV ECMO (RR 1.00 (CI 95% 0.78 to 1.30)), whereas those with two or fewer organ failures showed a substantial survival benefit RR 0.53 (CI 95% 0.36 to 0.78). Interpretation of this subgroup, however, is confounded by the high crossover rate to ECMO in the control arm of the larger trial, where rescue ECMO was permitted. This crossover likely reflects disease progression and a corresponding need for ECMO, complicating conclusions about the true effect of multiorgan failure on ECMO efficacy.

The emergence of COVID-19 during guideline development introduced a novel and substantial patient population with severe respiratory failure. Observational studies and registry data have provided important insights, demonstrating comparable outcomes to those seen in pre-pandemic RCTs.

In a large French registry study by Schmidt et al.^4,5^ the 60-day mortality among COVID-19 patients treated with VV-ECMO was 31%. Similarly, an international analysis from the Extracorporeal Life Support Organisation (ELSO) reported a 90-day hospital mortality of 30%, with central nervous system haemorrhage rate of 6%.^40^ Based on these findings, we do not recommend modifying our existing recommendation for VV ECMO in this patient subgroup. However, the resource-intensive nature of ECMO necessitates careful consideration of resource availability, especially during pandemic surges or in settings with constrained healthcare capacity.

The evidence supporting ECMO use in pregnant and peri-partum patients is limited, largely derived from case reports and small observational cohorts. Clinical complexity is increased by altered haemodynamics, haemostatic changes and the dual need for maternal and foetal care. Nonetheless, the available data suggest favourable outcomes.

A systematic review by Naoum et al.^41^ reported maternal survival rates of approximately 80% across all ECMO modalities, with foetal survival at 65%. In a separate analysis of the ELSO registry,^42^ found a maternal survival rate of 76.1% among patients supported with VV ECMO. While these results are encouraging, they must be interpreted cautiously due to potential publication bias and the heterogeneity of underlying conditions.

On balance, these findings do not justify altering our conditional recommendation for VV ECMO in this subgroup. However, implementation requires specialised care, including continuous foetal monitoring, tailored anticoagulation management, careful selection of pharmacotherapy and coordinated decisions regarding timing and mode of delivery.

Future research priorities

It is increasingly unlikely that further large-scale RCTs evaluating VV ECMO for severe hypoxic respiratory failure unresponsive to conventional therapy will be conducted. Similar to other acute and potentially fatal conditions, RCTs of life-support interventions such as ECMO face substantial ethical and logistical barriers. Challenges include maintaining clinical equipoise, addressing treatment failure and crossover and resolving instances where withdrawal of potentially lifesaving therapy is not feasible. These factors can compromise trial integrity and lead to inconclusive results, particularly when mortality is the primary outcome.

In light of these limitations, future research should prioritise the development and validation of robust mortality risk prediction tools. Accurate prognostication could help clinicians identify patients most likely to benefit from ECMO, improving patient selection and optimising resource utilisation. Furthermore, it remains an open question whether earlier or broader application of ECMO in patients with less severe hypoxemia could confer additional benefit, as is being investigated in the REDEEM Trial (NCT05562505). Evaluating ECMO in these lower-risk populations may require innovative trial designs, including adaptive, pragmatic or registry-based methodologies.

Additional priorities include defining optimal timing for ECMO initiation, refining criteria for weaning and understanding the long-term outcomes of survivors, including functional recovery and quality of life (EXCEL NCT03793257). Given the resource-intensive nature of ECMO and its growing utilisation, these efforts are critical to ensure evidence-based application and equitable access to care.

| **Summary of findings:** | | | | | | |
| --- | --- | --- | --- | --- | --- | --- |
| **VV-ECMO compared to usual care alone for adult patients with severe hypoxic respiratory failure** | | | | | | |
| **Patient or population:** adult patients with severe hypoxic respiratory failure  **Setting:** Intensive Care Units across Australia and New Zealand  **Intervention:** VV-ECMO  **Comparison:** usual care alone | | | | | | |
| Outcomes | **Anticipated absolute effects^*^** (95% CI) | | Relative effect (95% CI) | № of participants (studies) | Certainty of the evidence (GRADE) | Comments |
|  | **Risk with usual care alone** | **Risk with VV-ECMO** |  |  |  |  |
| 28 Day mortality | 409 per 1,000 | **233 per 1,000** (311 to 176) | **RR 0.57** (0.43 to 0.76) | 429 (2 RCTs)^1^ | ⨁⨁⨁◯ Moderate^a^ | In addition to conventional care, VV ECMO results in a reduction in short-term mortality (Day 28) in patients with severe hypoxic respiratory failure. |
| 90 Day mortality | 464 per 1,000 | **362 per 1,000** (468 to 283) | **RR 0.78** (0.61 to 1.01) | 368 (2 RCTs)^1^ | ⨁⨁⨁◯ Moderate^a^ | In addition to conventional care, VV ECMO reduces short term mortality (Day 90) slightly in patients with severe respiratory failure. |
| Neurological Injury | Stroke (ischaemic, hemorrhagic and massive GCS <8) reported in 2% (3/124) ECMO versus 6% (8/125) control.  Absolute risk difference -3.9 (CI 95%, -10.0 to 1.3) | |  | 251 (3 non-randomised studies)^2^ | ⨁◯◯◯ Very low^b,c,d^ | The evidence is very uncertain about the effect of VV ECMO in addition to conventional care on neurological Injury in survivors. |
| Quality of Life - SF-36  assessed with: SF-36 Scale from: 0 (worse) to 100 follow-up: range 6 months to 16 months | The mean quality of Life - SF-36 was **65.4** SF-36 | MD **5.4 SF-36 higher** (4.11 higher to 6.68 higher) | - | 245 (3 non-randomised studies)^3^ | ⨁⨁◯◯ Low | In addition to conventional care, VV ECMO is possibly associated with improved quality of life in survivors of severe hypoxic respiratory failure. |
| Long term mortality (1yr) | 607 per 1,000 | **426 per 1,000** (562 to 294) | **OR 0.48** (0.27 to 0.83) | 276 (2 non-randomised studies)^2^ | ⨁◯◯◯ Very low^a,e^ | In addition to conventional care, VV ECMO may reduce long term mortality (1yr) in patients with severe hypoxic respiratory failure but the evidence is very uncertain. |
| Cost per quality-adjusted life years  assessed with: AUD  follow-up: 6 months | Lifetime model would predict the cost per QALY of ECMO to be £19,255, (95% C.I. £7622-£59,200) AUD $35,258 (95% CI $13,956-$108,401)  ^f^ | |  | 180 (1 RCT)^4^ | ⨁⨁◯◯ Low^g,h,i,j^ | VV ECMO is likely associated with an acceptable cost per quality-adjusted life years . |
| Hospital Length of Stay  follow-up: range 1 days to 90 days | The mean hospital Length of Stay was **42.7** days | MD **14.05 days more** (8.59 more to 19.52 more) | - | 429 (2 RCTs)^1^ | ⨁⨁⨁◯ Moderate^a^ | In addition to conventional care, VV ECMO results in an increase in hospital length of stay in patients with severe hypoxic respiratory failure. |
| FEV1 follow-up: range 6 months to 12.5 months | The mean FEV1 was **79.5** percent | MD **2.58 percent lower** (15.59 lower to 10.43 higher) | - | 217 (2 non-randomised studies)^3^ | ⨁◯◯◯ Very low^d,k^ | The evidence is very uncertain about the effect of VV ECMO, in addition to conventional care on FEV1 in survivors. |
| FVC follow-up: range 6 months to 12.5 months | The mean FVC was **84.4** percent | MD **0.41 percent lower** (1.3 lower to 0.48 higher) | - | 217 (2 non-randomised studies)^3^ | ⨁◯◯◯ Very low^k^ | The evidence is very uncertain about the effect of VV ECMO, in addition to conventional care on FVC in survivors. |
| Depression  assessed with: HADS Scale from: 0 to 21 (worse) follow-up: range 6 months to 12.5 months | The mean Depression was **7.5** HADS | MD **1.6 HADS fewer** (1.8 fewer to 1.39 fewer) | - | 217 (2 non-randomised studies)^3^ | ⨁⨁◯◯ Low | In addition to conventional care, VV ECMO for severe hypoxic respiratory failure may be associated with a slight reduction in depression in survivors. |
| Anxiety  assessed with: HADS Scale from: 0 to 21 (worse) follow-up: range 6 months to 12.5 months | The mean Anxiety was **5.8** HADS | MD **1.3 HADS fewer** (2.01 fewer to 0.59 fewer) | - | 136 (2 non-randomised studies)^3^ | ⨁⨁◯◯ Low | In addition to conventional care, VV ECMO for severe hypoxic respiratory failure may be associated with a slight reduction in anxiety in survivors. |
| Quality of Life Physical Component Score (HRQOL ) assessed with: PCS Scale from: 0 (worse) to 100 follow-up: range 6 months to 16 months | The mean quality of Life Physical Component Score was **34.9** PCS | MD **3.13 PCS higher** (2.44 higher to 3.83 higher) | - | 245 (3 non-randomised studies)^3^ | ⨁⨁◯◯ Low | In addition to conventional care, VV ECMO is possibly associated with less physical disability in survivors of severe hypoxic respiratory failure. |
| Quality of Life - Mental Component Score (QOL ) assessed with: MCS Scale from: 0 (worse) to 100 follow-up: range 6 months to 16 months | The mean quality of Life - Mental Component Score was **38.3** MCS | MD **2.34 MCS higher** (1.72 higher to 2.96 higher) | - | 245 (3 non-randomised studies)^3^ | ⨁⨁◯◯ Low | In addition to conventional care, VV ECMO is possibly associated with less mental disability in survivors of severe hypoxic respiratory failure. |
| ***The risk in the intervention group** (and its 95% confidence interval) is based on the assumed risk in the comparison group and the **relative effect** of the intervention (and its 95% CI).  **CI:** confidence interval; **MD:** mean difference; **OR:** odds ratio; **RR:** risk ratio | | | | | | |
| **GRADE Working Group grades of evidence** **High certainty:** we are very confident that the true effect lies close to that of the estimate of the effect. **Moderate certainty:** we are moderately confident in the effect estimate: the true effect is likely to be close to the estimate of the effect, but there is a possibility that it is substantially different. **Low certainty:** our confidence in the effect estimate is limited: the true effect may be substantially different from the estimate of the effect. **Very low certainty:** we have very little confidence in the effect estimate: the true effect is likely to be substantially different from the estimate of effect. | | | | | | |

#### Explanations

a. Does not meet OIS criteria

b. Concerns over deviation from intended intervention, however appropriate analysis was used to estimate the effect; decision not to rate down

c. Includes both appreciable benefit and appreciable harm

d. Substantial heterogeneity

e. Evidence not provided for critical appraisal or publication bias; lacking appropriate directives for practice

f. GBP from 2009 conversion to AUD November 2021 rate 1.83

g. only one study

h. Different health care system (UK)

i. Wide CI however, well within the range regarded as cost effective by health technology assessment organisations

j. OIS could not be calculated with information provided

k. Confidence interval crosses the line of no effect

References

1. International Guidelines Library [Internet]. GIN. [cited 2023 Feb 20]. Available from: https://g-i-n.net/international-guidelines-library

2. ECRI Guidelines Trust® [Internet]. ECRI. [cited 2023 Feb 20]. Available from: https://d84vr99712pyz.cloudfront.net/p/images1/ecri-trusted-voice-healthcare.jpg

3. EtD’s and Guidelines [Internet]. [cited 2023 Feb 20]. Available from: https://guidelines.gradepro.org/search

4. MAGICapp - Making GRADE the Irresistible Choice - Guidelines and Evidence summaries [Internet]. [cited 2023 Feb 20]. Available from: https://app.magicapp.org/#/guidelines

5. BIGG-REC - GRADE Recommendations [Internet]. [cited 2023 Feb 20]. Available from: https://bigg-rec.bvsalud.org/en

6. Find guidance [Internet]. NICE. NICE; [cited 2023 Feb 20]. Available from: https://www.nice.org.uk/guidance

7. ELSO Guidelines | Extracorporeal Membrane Oxygenation (ECMO) [Internet]. [cited 2023 Feb 20]. Available from: https://www.elso.org/ecmo-resources/elso-ecmo-guidelines.aspx

8. Trip Medical Database [Internet]. [cited 2023 Feb 20]. Available from: https://www.tripdatabase.com/

9. Overview | Extracorporeal membrane oxygenation for severe acute respiratory failure in adults | Guidance | NICE [Internet]. NICE; 2011 [cited 2024 Sept 21]. Available from: https://www.nice.org.uk/guidance/ipg391

10. Guidelines on the Management of Acute Respiratory Distress Syndrome (ARDS). Fac Intensive Care Med [Internet]. 2018; Available from: https://www.ficm.ac.uk/sites/ficm/files/documents/2021-10/Guidelines_on_the_Management_of_Acute_Respiratory_Distress_Syndrome.pdf

11. Fan E, Del Sorbo L, Goligher EC, Hodgson CL, Munshi L, Walkey AJ, et al. An Official American Thoracic Society/European Society of Intensive Care Medicine/Society of Critical Care Medicine Clinical Practice Guideline: Mechanical Ventilation in Adult Patients with Acute Respiratory Distress Syndrome. Am J Respir Crit Care Med. 2017 May 1;195(9):1253–63.

12. Fichtner F, Moerer O, Laudi S, Weber-Carstens S, Nothacker M, Kaisers U, et al. Mechanical Ventilation and Extracorporeal Membrane Oxygena tion in Acute Respiratory Insufficiency. Dtsch Arzteblatt Int. 2018 Dec 14;115(50):840–7.

13. Peek GJ, Clemens F, Elbourne D, Firmin R, Hardy P, Hibbert C, et al. CESAR: conventional ventilatory support vs extracorporeal membrane oxygenation for severe adult respiratory failure. BMC Health Serv Res. 2006 Dec 23;6:163.

14. Combes A, Hajage D, Capellier G, Demoule A, Lavoué S, Guervilly C, et al. Extracorporeal Membrane Oxygenation for Severe Acute Respiratory Distress Syndrome. N Engl J Med. 2018 May 24;378(21):1965–75.

15. Klugar M, Lotfi T, Darzi AJ, Reinap M, Klugarová J, Kantorová L, et al. GRADE Guidance 39: Using GRADE-ADOLOPMENT to adopt, adapt or create contextualized recommendations from source guidelines and evidence syntheses. J Clin Epidemiol [Internet]. 2024 Aug 5 [cited 2024 Aug 8];0(0). Available from: https://www.jclinepi.com/article/S0895-4356(24)00250-6/abstract

16. Epistemonikos: Database of the best Evidence-Based Health Care [Internet]. [cited 2023 Feb 20]. Available from: https://www.epistemonikos.org/

17. National Institute for Health Research. PROSPERO: international prospective register of systematic reviews. [Internet]. International prospective register of systematic reviews. [cited 2023 Jan 20]. Available from: https://www.crd.york.ac.uk/prospero/

18. Critical Appraisal Tools | JBI [Internet]. [cited 2023 Feb 20]. Available from: https://jbi.global/critical-appraisal-tools

19. Combes A, Peek GJ, Hajage D, Hardy P, Abrams D, Schmidt M, et al. ECMO for severe ARDS: systematic review and individual patient data meta-analysis. Intensive Care Med. 2020 Nov;46(11):2048–57.

20. Mendes PV, Melro LMG, Li HY, Joelsons D, Zigaib R, Ribeiro JM da FP, et al. Extracorporeal membrane oxygenation for severe acute respiratory distress syndrome in adult patients: a systematic review and meta-analysis. Rev Bras Ter Intensiva. 2019;31(4):548–54.

21. Munshi L, Walkey A, Goligher E, Pham T, Uleryk EM, Fan E. Venovenous extracorporeal membrane oxygenation for acute respiratory distress syndrome: a systematic review and meta-analysis. Lancet Respir Med. 2019 Feb;7(2):163–72.

22. Vaquer S, de Haro C, Peruga P, Oliva JC, Artigas A. Systematic review and meta-analysis of complications and mortality of veno-venous extracorporeal membrane oxygenation for refractory acute respiratory distress syndrome. Ann Intensive Care. 2017 Dec;7(1):51.

23. Wang J, Wang Y, Wang T, Xing X, Zhang G. Is Extracorporeal Membrane Oxygenation the Standard Care for Acute Respiratory Distress Syndrome: A Systematic Review and Meta-Analysis. Heart Lung Circ. 2021 May;30(5):631–41.

24. Wilcox ME, Jaramillo-Rocha V, Hodgson C, Taglione MS, Ferguson ND, Fan E. Long-Term Quality of Life After Extracorporeal Membrane Oxygenation in ARDS Survivors: Systematic Review and Meta-Analysis. J Intensive Care Med. 2020 Mar;35(3):233–43.

25. Zhu Y, Zhang M, Zhang R, Ye X, Wei J. Extracorporeal membrane oxygenation versus mechanical ventilation alone in adults with severe acute respiratory distress syndrome: A systematic review and meta-analysis. Int J Clin Pract. 2021 Sept;75(9):e14046.

26. MedlinePlus - Health Information from the National Library of Medicine [Internet]. [cited 2023 Feb 20]. Available from: https://medlineplus.gov/

27. Welcome - Embase [Internet]. [cited 2023 Feb 20]. Available from: https://www.embase.com/landing?status=grey

28. Search | Cochrane Library [Internet]. [cited 2023 Feb 20]. Available from: https://www.cochranelibrary.com/central

29. Muñoz J, Santa-Teresa P, Tomey MJ, Visedo LC, Keough E, Barrios JC, et al. Extracorporeal membrane oxygenation (ECMO) in adults with acute respiratory distress syndrome (ARDS): A 6-year experience and case-control study. Heart Lung J Crit Care. 2017;46(2):100–5.

30. Peek GJ, Mugford M, Tiruvoipati R, Wilson A, Allen E, Thalanany MM, et al. Efficacy and economic assessment of conventional ventilatory support versus extracorporeal membrane oxygenation for severe adult respiratory failure (CESAR): a multicentre randomised controlled trial. Lancet Lond Engl. 2009 Oct 17;374(9698):1351–63.

31. Sterne JAC, Savović J, Page MJ, Elbers RG, Blencowe NS, Boutron I, et al. RoB 2: a revised tool for assessing risk of bias in randomised trials. BMJ. 2019 Aug 28;366:l4898.

32. Cochrane Handbook for Systematic Reviews of Interventions (current version) | Cochrane [Internet]. [cited 2025 Oct 21]. Available from: https://www.cochrane.org/authors/handbooks-and-manuals/handbook/current

33. Griffiths M, Maruszczak M, Kusel J. The who-choice cost-effectiveness Threshold: a Country-level analysis of changes over time. Value Health. 2015 May 1;18(3):A88.

34. Alonso-Coello P, Schünemann HJ, Moberg J, Brignardello-Petersen R, Akl EA, Davoli M, et al. GRADE Evidence to Decision (EtD) frameworks: a systematic and transparent approach to making well informed healthcare choices. 1: Introduction. BMJ. 2016 June 28;353:i2016.

35. Schünemann HJ, Wiercioch W, Brozek J, Etxeandia-Ikobaltzeta I, Mustafa RA, Manja V, et al. GRADE Evidence to Decision (EtD) frameworks for adoption, adaptation, and de novo development of trustworthy recommendations: GRADE-ADOLOPMENT. J Clin Epidemiol. 2017 Jan 1;81:101–10.

36. Higgins AM, Pettilä V, Harris AH, Bailey M, Lipman J, Seppelt IM, et al. The critical care costs of the influenza A/H1N1 2009 pandemic in Australia and New Zealand. Anaesth Intensive Care. 2011 May;39(3):384–91.

37. Oude Lansink-Hartgring A, Miranda DDR, Mandigers L, Delnoij T, Lorusso R, Maas JJ, et al. Health-related quality of life, one-year costs and economic evaluation in extracorporeal membrane oxygenation in critically ill adults. J Crit Care. 2023 Feb 1;73:154215.

38. O’Neill J, Tabish H, Welch V, Petticrew M, Pottie K, Clarke M, et al. Applying an equity lens to interventions: using PROGRESS ensures consideration of socially stratifying factors to illuminate inequities in health. J Clin Epidemiol. 2014 Jan 1;67(1):56–64.

39. Schmidt M, Hajage D, Lebreton G, Monsel A, Voiriot G, Levy D, et al. Extracorporeal membrane oxygenation for severe acute respiratory distress syndrome associated with COVID-19: a retrospective cohort study. Lancet Respir Med. 2020 Nov;8(11):1121–31.

40. Barbaro RP, MacLaren G, Boonstra PS, Iwashyna TJ, Slutsky AS, Fan E, et al. Extracorporeal membrane oxygenation support in COVID-19: an international cohort study of the Extracorporeal Life Support Organization registry. Lancet Lond Engl. 2020 Oct 10;396(10257):1071–8.

41. Naoum EE, Chalupka A, Haft J, MacEachern M, Vandeven CJM, Easter SR, et al. Extracorporeal Life Support in Pregnancy: A Systematic Review. J Am Heart Assoc. 2020 July 7;9(13):e016072.

42. Ramanathan K, Tan CS, Rycus P, Anders M, Lorusso R, Zhang JJY, et al. Extracorporeal Membrane Oxygenation in Pregnancy: An Analysis of the Extracorporeal Life Support Organization Registry. Crit Care Med. 2020 May;48(5):696–703.

**Core question 2**

**In the management of adult patients with severe hypercapnic respiratory failure, should VV ECMO or usual care alone be used?**

Literature search and evaluation

Phase 1 search for existing guidelines, eight guideline portals (GIN^1^, ECRI Guidelines Trust^2^, Database of GRADE EtD’s and Guidelines^3^, MAGICapp^4^, BIGG International database of GRADE guidelines^5^, NICE guidelines^6^, ELSO guidelines^7^, TRIP database^8^ were searched using the search string “extracorporeal membrane oxygenation” and “ECMO”. The inclusion period was from inception up to September 2023. 134 references were imported into Covidence Software^9^ for screening.


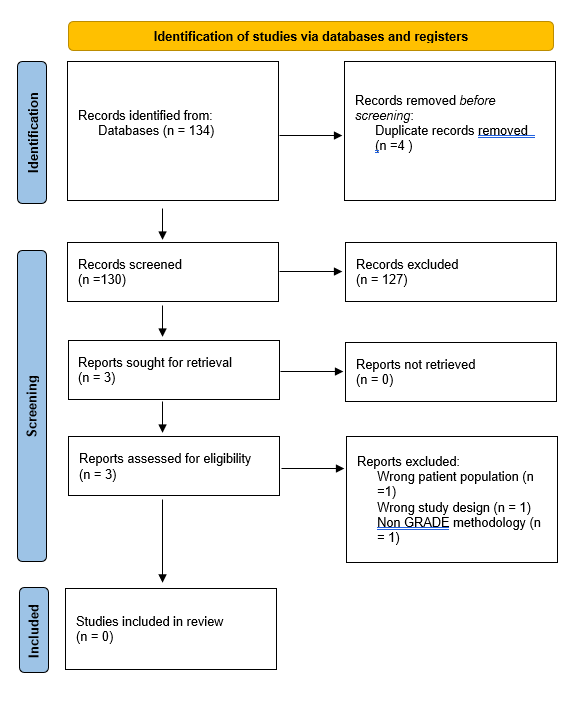


Figure 1. PRISMA flow chart of guidelines for core question 2

The Phase 2 search for existing systematic reviews related to core question 2 was completed using two systematic review databases, Epistemonikos^10^ and PROSPERO^11^, and employed the search string “extracorporeal membrane oxygenation OR ECMO AND asthma* OR COPD OR chronic obstructive pulmonary disease OR hypercap* OR hypercarb*”. The inclusion period extended from inception to September 2023.


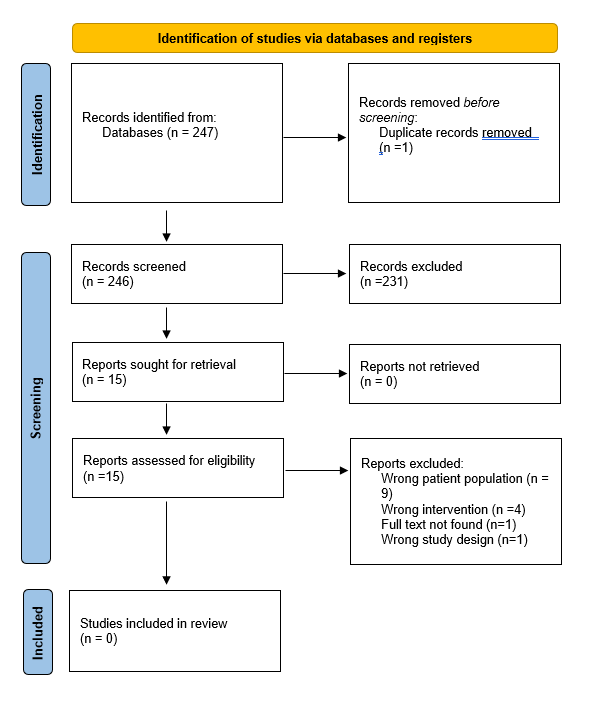


Figure 2. PRISMA flow chart of systematic reviews for core question 2

At the outset of our evidence search, we included studies involving patients with secondary hypercapnia, for example, those with ARDS where hypercapnia is a consequence of hypoventilation due to severe lung injury. These studies often evaluated the use of VV ECMO as a rescue strategy for global respiratory failure, which included hypercapnia as part of the broader syndrome. However, as our review progressed it became clear that the clinical question the GDG were most interested in focused on primary hypercapnic respiratory failure, specifically populations such as those with acute exacerbations of chronic obstructive pulmonary disease (COPD) or status asthmaticus. These conditions are characterised by ventilatory failure with preserved oxygenation, where hypercapnia and acidosis are the principal indications for escalated support. Accordingly, we refined our inclusion criteria to focus on studies evaluating the role of VV ECMO where the therapeutic goal of ECMO is primarily to facilitate carbon dioxide removal and correct respiratory acidosis rather than improve oxygenation. We identified a subset of studies evaluating pumpless extracorporeal carbon dioxide removal (ECCO_2_R) devices, but as our focus is on pump-driven VV ECMO, these studies were excluded from our analysis.

Phase 3 of the search for primary literature was completed systematically, searching two databases, Pubmed^12^ and Embase^13^ using the following search strings. Inclusion was from inception (PubMed) and 1996 (Embase) to September 2023.

(extracorporeal membrane oxygenation OR ECMO OR VV-ecmo OR veno-venous extracorporeal membrane oxygenation OR venovenous extracorporeal membrane oxygenation OR extracorporeal oxygenation OR veno-venous extracorporeal oxygenation) AND (((asthma*) OR (COPD OR chronic obstructive pulmonary disease)) OR (hypercap* OR hypercarb*))

Embase 1996 to September 2023

1 exp extracorporeal oxygenation/

2 veno-venous ECMO/

3 venovenous extracorporeal membrane oxygenation.mp.

4 veno-venous extracorporeal membrane oxygenation.mp.

5 VV-ecmo.mp.

6 VV ecmo.mp.

7 VV extracorporeal membrane oxygenation.mp.

8 1 or 2 or 3 or 4 or 5 or 6 or 7

9 hypercapnia/

10 asthma*.mp. [mp=title, abstract, heading word, drug trade name, original title, device manufacturer, drug manufacturer, device trade name, keyword heading word, floating subheading word, candidate term word]

11 copd.mp. [mp=title, abstract, heading word, drug trade name, original title, device manufacturer, drug manufacturer, device trade name, keyword heading word, floating subheading word, candidate term word]

12 chronic obstructive pulmonary disease.mp. [mp=title, abstract, heading word, drug trade name, original title, device manufacturer, drug manufacturer, device trade name, keyword heading word, floating subheading word, candidate term word]

13 9 or 10 or 11 or 12

14 8 and 13


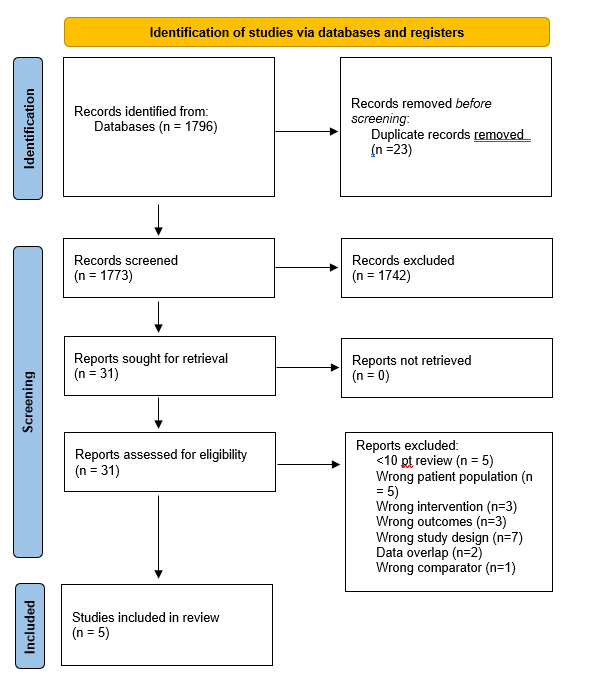


Figure 3. PRISMA flow chart of primary literature for core question 2.

| **Study** | **Design** | **Population** | **Intervention/**  **Comparison** | **Key Outcomes** | **Limitations** |
| --- | --- | --- | --- | --- | --- |
| Zakrajsek et al., 2023^14^ | Retrospective multicentre cohort (US) | 13,714 patients with asthma exacerbation requiring IMV (2010-2020); 127 received ECMO | ECMO (VV, VA, VAV) vs. usual care | Adjusted models: ECMO associated with reduced mortality (OR range: 0.33-0.48)  Unadjusted: ECMO associated with higher mortality (NS) | No ECMO mode stratification;  Observational design;  Possible residual confounding |
| Warren et al., 2020^15^ | Retrospective cohort (UK ECMO registry) | Patients with life-threatening asthma vs. other indications, all receiving VV ECMO (2011-2017) | Descriptive comparison of asthma vs. non-asthma VV ECMO recipients | Lower ICU mortality for asthma (4.6% vs 28%)  Shorter ECMO duration (186 vs 302hrs) | No comparator group; unpublished subgroup data;  Limited external validity |
| Yeo et al., 2017^16^ | Retrospective cohort (ELSO registry) | 250 patients with severe asthma on VV ECMO (1192-2016) | No comparison group | 16.4% in-hospital mortality | No comparison group;  Historical dataset;  Potential selection bias |
| Di Lascio et al., 2017^17^ | Case series | 13 patients with status asthmaticus receiving VV ECMO | No comparison group | 0% mortality | Small sample size;  Single centre;  No comparison group |
| Vutipongsatorn et al., 2019^18^ | Case series | 10 patients with life-threatening asthma on VV ECMO | No comparison group | 0% mortality | Very small sample size; retrospective;  No comparison group; limited generalisabilty |

^VA: veno-arterial; VAV: veno-arterial-venous;^

Table 1. Summary of primary literature considered for core question 2

| **Study** | **Confounding** | **Selection of Participants** | **Classification of Interventions** | **Deviations from Intended Interventions** | **Missing Data** | **Outcome Measurement** | **Selective Reporting** | **Overall** |
| --- | --- | --- | --- | --- | --- | --- | --- | --- |
| **Zakrajsek et al., 2023** | 🟠 | 🟡 | 🟡 | 🟢 | 🟡 | 🟢 | 🟡 | 🟠 |
| **Warren et al., 2020** | 🔴 | 🟡 | 🟢 | 🟢 | ⚪ | 🟢 | 🔴 | 🔴 |
| **Yeo et al., 2017** | 🔴 | 🟡 | 🟢 | 🟢 | 🟡 | 🟢 | 🟡 | 🔴 |
| **Di Lascio et al., 2017** | 🔴 | 🟠 | 🟢 | 🟢 | 🟢 | 🟢 | 🟡 | 🔴 |
| **Vutipongsatorn et al., 2019** | 🔴 | 🟠 | 🟢 | 🟢 | 🟢 | 🟢 | 🟡 | 🔴 |

^ROBINS-I Key: 🟢 low risk 🟡moderate risk 🟠serious risk 🔴critical risk ⚪no information^

Table 2. ROBINS-I Appraisal of primary papers used in core question 2

Synthesis of evidence

No RCTs have directly compared VV ECMO to usual care in patients with severe hypercapnic respiratory failure, and thus no high-certainty evidence exists to inform the balance of benefits and harms in this population. However, indirect evidence from observational studies and registry data provides limited insights into potential undesirable effects associated with VV ECMO.

Zakrajsek et al. (2023) analysed a large retrospective cohort of patients with acute asthma requiring invasive MV.^14^ A propensity score-matched subgroup analysis (82 of 127 ECMO patients) found that ECMO was associated with increased rates of gastrointestinal and respiratory haemorrhage, cardiac arrythmias and infections. However, adverse events were rare, and ECMO patients experienced lower rates of neurological complications, including intracranial haemorrhage and cardiac arrest. Interpretation of these findings is limited by methodological concerns, including the inclusion of all ECMO modalities (VA, VAV), lack of transparent matching procedures and exclusion of over one-third of ECMO patients from the matched analysis. Authors were contacted for data allowing isolation of the VV ECMO subgroup.

Broader registry data, such as from the EXCEL registry^19^, report a high rate of new disability among survivors of VV ECMO across all indications. In this cohort (n=135), 35% of survivors experienced new disability, and mortality was 27%. While this reflects outcomes across diverse indications rather than specifically hypercapnic respiratory failure, it underscores the importance of weighing functional outcomes and survivorship burden in ECMO decision-making.

Although derived from a different population and intervention, the REST trial^20^ reported increased adverse events in patients with acute hypoxic respiratory failure randomised to low-flow extracorporeal CO_2_ removal. Serious complications included intracranial haemorrhage and infectious events. These findings highlight the potential for device-related harms across extracorporeal support platforms and provide an indirect safety signal.

In the absence of direct comparative data in the population of interest, the potential for serious but infrequent complications associated with VV ECMO must be considered alongside uncertain benefit. No data exists evaluating whether VV ECMO facilitates lung-protective ventilation in hypercapnic respiratory failure.

Certainty of Evidence

Important concerns about risk of bias, indirectness and imprecision were present across all available evidence. Additionally, many studies lack appropriate comparator groups, precluding direct estimation of treatment effect. Given these limitations, no reliable conclusion can be drawn about the benefits or harms of VV ECMO compared to usual care in this population. As a result, no recommendation for or against the use of VV ECMO can be made based on current evidence, and further research is required to address this critical evidence gap.

Future Research Priorities

Due to the low incidence of severe hypercapnic respiratory failure meeting criteria for VV ECMO, conducting adequately powered RCTs may be challenging. Barriers include low patient volumes, heterogeneity in patient selection and ethical concerns about equipoise in life-threatening situations. Ideally, well-designed multicentre randomised trials or prospective observational studies with appropriate comparator groups to assess the effectiveness and safety of VV ECMO in this population, particularly in conditions such as life-threatening asthma or COPD exacerbations. These studies should aim to include detailed reporting of adverse events, functional outcomes and quality of life in survivors. Additionally, studies are needed to determine whether VV ECMO mitigates ventilator-induced lung injury in hypercapnic patients.

While RCTs remain the gold standard for minimising bias and confounding, in this setting, their feasibility is limited. Therefore, future research may be more pragmatically advanced through the use of high-quality comparative observational studies, particularly those that leverage large multicentre or national datasets. Techniques such as propensity score matching can help address confounding and emulate trial conditions. This approach relies on the availability of detailed high-quality clinical data, such as is provided in the binational ECMO registry EXCEL (NCT03793257). Embedding prospective cohort studies within these registries or designing adaptive platform trials could offer a practical compromise between rigour and feasibility. In summary, while RCTs are ideal, large-scale, methodologically rigorous observational research is likely to be the most feasible and informative path forward in this rare but critical clinical scenario.

References

1. International Guidelines Library [Internet]. GIN. [cited 2023 Feb 20]. Available from: https://g-i-n.net/international-guidelines-library

2. ECRI Guidelines Trust® [Internet]. ECRI. [cited 2023 Feb 20]. Available from: https://d84vr99712pyz.cloudfront.net/p/images1/ecri-trusted-voice-healthcare.jpg

3. EtD’s and Guidelines [Internet]. [cited 2023 Feb 20]. Available from: https://guidelines.gradepro.org/search

4. MAGICapp - Making GRADE the Irresistible Choice - Guidelines and Evidence summaries [Internet]. [cited 2023 Feb 20]. Available from: https://app.magicapp.org/#/guidelines

5. BIGG-REC - GRADE Recommendations [Internet]. [cited 2023 Feb 20]. Available from: https://bigg-rec.bvsalud.org/en

6. Find guidance [Internet]. NICE. NICE; [cited 2023 Feb 20]. Available from: https://www.nice.org.uk/guidance

7. ELSO Guidelines | Extracorporeal Membrane Oxygenation (ECMO) [Internet]. [cited 2023 Feb 20]. Available from: https://www.elso.org/ecmo-resources/elso-ecmo-guidelines.aspx

8. Trip Medical Database [Internet]. [cited 2023 Feb 20]. Available from: https://www.tripdatabase.com/

9. Covidence - Better systematic review management [Internet]. [cited 2024 Aug 28]. Available from: https://www.covidence.org/

10. Epistemonikos: Database of the best Evidence-Based Health Care [Internet]. [cited 2023 Feb 20]. Available from: https://www.epistemonikos.org/

11. PROSPERO: International prospective register of systematic reviews [Internet]. PROSPERO. [cited 2023 Feb 20]. Available from: https://www.crd.york.ac.uk/prospero/

12. PubMed [Internet]. PubMed. [cited 2024 Sept 13]. Available from: https://pubmed.ncbi.nlm.nih.gov/

13. Welcome - Embase [Internet]. [cited 2023 Feb 20]. Available from: https://www.embase.com/landing?status=grey

14. Zakrajsek JK, Min SJ, Ho PM, Kiser TH, Kannappan A, Sottile PD, et al. Extracorporeal Membrane Oxygenation for Refractory Asthma Exacerbations With Respiratory Failure. Chest. 2023 Jan 1;163(1):38–51.

15. Warren, A W, Yd C, Ss V, Ja F, N S, J B, et al. Outcomes of the NHS England National Extracorporeal Membrane Oxygenation Service for adults with respiratory failure: a multicentre observational cohort study. Br J Anaesth [Internet]. 2020 Sept [cited 2025 Feb 14];125(3). Available from: https://pubmed.ncbi.nlm.nih.gov/32736826/

16. Yeo HJ, Kim D, Jeon D, Kim YS, Rycus P, Cho WH. Extracorporeal membrane oxygenation for life-threatening asthma refractory to mechanical ventilation: analysis of the Extracorporeal Life Support Organization registry. Crit Care Lond Engl. 2017 Dec 6;21(1):297.

17. Di Lascio G, Prifti E, Messai E, Peris A, Harmelin G, Xhaxho R, et al. Extracorporeal membrane oxygenation support for life-threatening acute severe status asthmaticus. Perfusion. 2017 Mar;32(2):157–63.

18. Vutipongsatorn K, Fujitake E, Singh S. Extracorporeal membrane oxygenation in life-threatening asthma unresponsive to mechanical ventilation: a comparison of patient demographics and outcomes between a large London-based intensive care unit and an international registry. Clin Med. 2019 June;19(Suppl 3):19.

19. Hodgson CL, Higgins AM, Bailey MJ, Anderson S, Bernard S, Fulcher BJ, et al. Incidence of death or disability at 6 months after extracorporeal membrane oxygenation in Australia: a prospective, multicentre, registry-embedded cohort study. Lancet Respir Med. 2022 Nov;10(11):1038–48.

20. McNamee JJ, Gillies MA, Barrett NA, Perkins GD, Tunnicliffe W, Young D, et al. Effect of Lower Tidal Volume Ventilation Facilitated by Extracorporeal Carbon Dioxide Removal vs Standard Care Ventilation on 90-Day Mortality in Patients With Acute Hypoxemic Respiratory Failure: The REST Randomized Clinical Trial. JAMA. 2021 Sept 21;326(11):1013–23.

**Core question 3**

**In the management of adult patients with ARDS receiving VV ECMO, should prone positioning or usual care alone be used?**

Literature search and evaluation

Phase 1 search for existing guidelines, eight guideline portals (GIN^1^, ECRI Guidelines Trust^2^, Database of GRADE EtD’s and Guidelines^3^, MAGICapp^4^, BIGG International database of GRADE guidelines^5^, NICE guidelines^6^, ELSO guidelines^7^, TRIP database^8^ were searched using the search string “extracorporeal membrane oxygenation” and “ECMO”. The inclusion period was from inception up to September 2023.


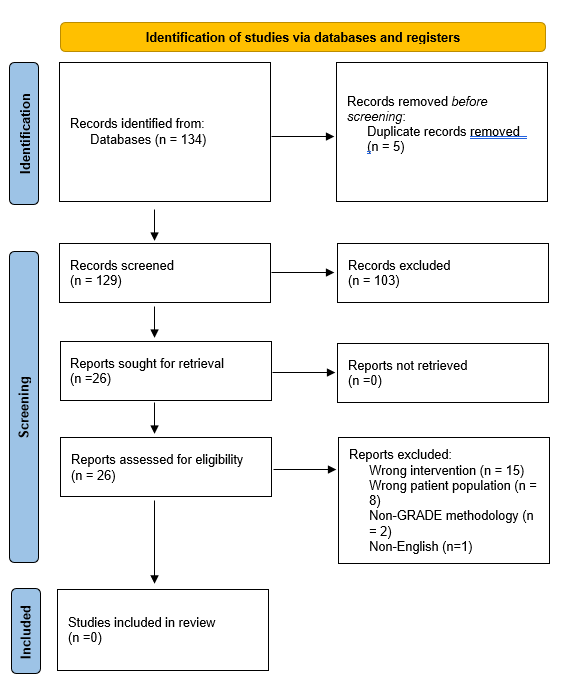


Figure 1. PRISMA flow chart for guidelines for core question 3

Phase 2 search for existing systematic reviews for core question 3 was completed using two systematic review databases, Epitemonikos^9^ and PROSPERO^10^ and the search strings “extracorporeal membrane oxygenation” or “ECMO”. The inclusion was from March 2021 to January 2024. Forty-four references were imported for screening, eight of which addressed the relevant population and intervention, however, it was decided not to include these studies as none of them included the most recent RCT PRONECMO. It was decided that the evidence team would complete an update to the published MA by authors Poon et al.^11^

Systematic searches of the following databases (PubMed,^12^ Cochrane,^13^ Embase^14^ and Scopus^15^) were completed, to replicate the original MA. The inclusion dates were March 2021 to January 2024.

PubMed

1. “extracorporeal membrane oxygenation” [MeSH]
2. (ECMO [title/abstract] OR “extracorporeal membrane oxygenat*” [title/abstract] OR “extracorporeal life support*” [title/abstract] OR ECLS [title/abstract] OR “membrane oxygenat*” [title/abstract])
3. #1 OR #2
4. “prone position” [MeSH]
5. (“prone position*” [title/abstract] OR pron* [title/abstract] OR “positioning therapy” [title/abstract])
6. #4 OR #5
7. #3 AND #6 (“prone position”[MeSH Terms] OR (“prone position”[title/abstract] OR “pron*”[ title/abstract])) AND (((((“ECMO” [title/abstract] OR “extracorporeal membrane oxygenat*”[title/abstract]) OR “extracorporeal life support” [title/abstract]) OR “ECLS”[title/abstract]) OR “membrane oxygenat*”[title/abstract]) OR “extracorporeal membrane oxygenation”[MeSH Terms])

Cochrane

1. MeSH descriptor: [extracorporeal membrane oxygenation] explode all trees
2. (ECMO OR “Extracorporeal membrane oxygenat*” OR “Extracorporeal Life Support*” OR ECLS OR “membrane oxygenat*”):ti,ab,kw
3. MeSH descriptor: [prone position] explode all trees
4. (“prone position*” OR pron* OR “positioning therapy”):ti,ab,kw
5. (#1 OR #2) AND (#3 OR #4)

Embase

1. 'extracorporeal oxygenation'/exp OR 'ecmo':ab,kw,ti OR 'ecls':ab,kw,ti OR 'extracorporeal membrane oxygenat*':ab,kw,ti OR 'extracorporeal life support*':ab,kw,ti OR 'membrane oxygenat*':ab,kw,ti
2. 'prone position'/exp OR 'prone position*':ab,ti,kw OR 'pron*':ab,ti,kw OR ‘positioning therapy’:ab,ti,kw
3. #1 AND #2
4. #1 AND #2 AND [English/lim AND [article]/lim

Scopus

1. TITLE-ABS-KEY(“Extracorporeal membrane oxygenat*” OR “ECMO” OR “ECLS” OR “Extracorporeal life support*” OR “membrane oxygenat*”)
2. TITLE-ABS-KEY(“pron* position*” OR “pron*” OR “positioning therapy”)
3. #1 AND #2
4. #3 lim:english, Lim: article


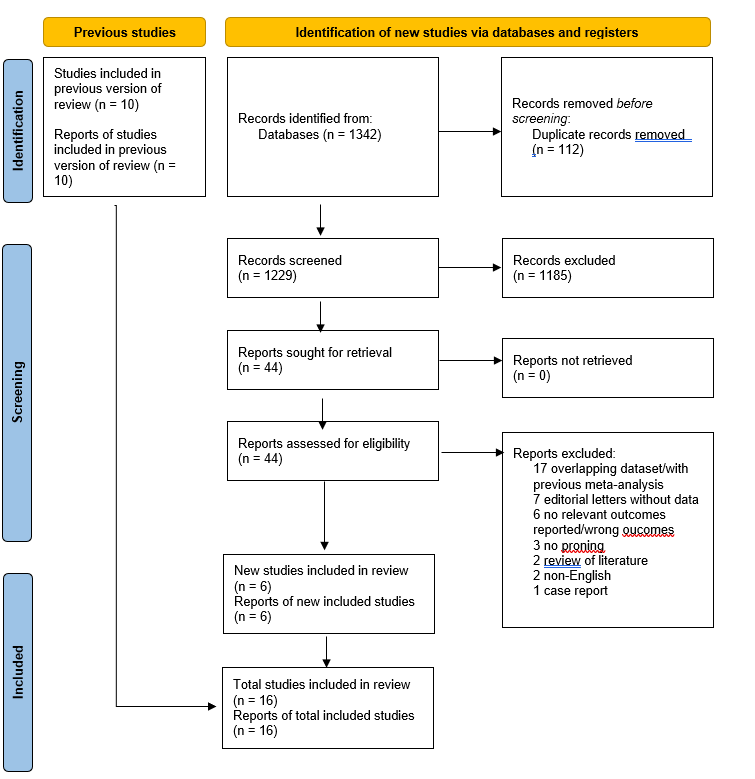


Figure 2. PRISMA flow chart of primary literature included in the updated MA for core question 3.

The evidence team conducted a SR and MA to evaluate the efficacy and safety of prone positioning compared with usual care in adult patients with ARDS receiving VV ECMO. This study represents an update to the MA by Poon et al.^11^ For this MA, we included studies published in English that reported adult patients (≥18 years) supported with ECMO for ARDS, in which prone positioning was explicitly described and associated outcomes were clearly reported. We excluded studies using ELSO registry data (to avoid data duplication), cohorts with fewer than ten patients, non-human studies, reviews and case reports. Six new studies and ten studies from the previous analysis were included. Some studies were excluded due to data overlap. There were a total of 1448 patients, 698 for whom the prone position was used whilst the patient was on VV ECMO and 750 patients for whom VV ECMO alone was used. The risk of bias was assessed independently by two reviewers using validated tools appropriate to the study design. For RCTs, the RoB 2^16^ and non-randomised studies the Newcastle-Ottawa Scale (NOS) was used.^17^

| **Title** | Author | Year | Outcomes addressed | Population |
| --- | --- | --- | --- | --- |
| Beneficial Effect of Prone Positioning During Venovenous Extracorporeal Membrane Oxygenation for Coronavirus Disease 2019^18^ | Zaaqoq et al. | 2022 | MV duration | ARDS (COVID-19) pts on VV ECMO (n=232)  PP =67 |
| Extracorporeal membrane oxygenation for SARS-CoV-2 acute respiratory distress syndrome: a retrospective study from Hubei, China^19^ | Yang et al. | 2021 | ECMO duration | ARDS (COVID-19) pts on VV ECMO (n=73)  PP= 51 |
| Effect of prone position in patients with acute respiratory distress syndrome supported by venovenous extracorporeal membrane oxygenation: a retrospective cohort study^20^ | Chen et al. | 2022 | Mortality (at longest follow-up)  ECMO duration  ICU LOS | ARDS pts on VV ECMO (n=91)  PP=38 |
| Prone positioning monitored by electrical impedance tomography in patients with severe acute respiratory distress syndrome on veno-venous ECMO^21^ | Franchineau et al. | 2020 | Mortality (at longest follow-up)  MV duration  ECMO duration  ICU LOS | ARDS pts on VV ECMO (n=21)  PP= 21 |
| Prone positioning and extracorporeal membrane oxygenation for severe acute respiratory distress syndrome: time for a randomized trial?^22^ | Guervilly et al. | 2019 | Long-term mortality (>90 days)  ECMO duration  MV duration | ARDS pts on VV ECMO (n=168)  PP=107 |
| Prone Positioning During Extracorporeal Membrane Oxygenation in Patients With Severe ARDS: The PRONECMO Randomized Clinical Trial^23^ | Schmidt et al. | 2023 | Mortality (at longest follow-up)  ECMO duration  MV duration | ARDS pts on VV ECMO (n=170)  PP=86 |
| Impact of Prone Position in COVID-19 Patients on Extracorporeal Membrane Oxygenation^24^ | Massart et al. | 2023 | Mortality (at longest follow-up)  ECMO duration  MV duration | ARDS (COVID-19) pts on VV ECMO (n=517)  PP=364 |
| Prolonged prone positioning under VV ECMO is safe and improves oxygenation and respiratory compliance^25^ | Kimmoun et al. | 2015 | ECMO duration | ARDS pts on VV ECMO (n=44)  PP=17 |
| Prone positioning under VV ECMO in SARS-CoV-2-induced acute respiratory distress syndrome^26^ | Garcia et al. | 2020 | ECMO duration | ARDS (COVID-19) pts on VV ECMO (n=25)  PP=14 |
| Prone-Positioning for Severe Acute Respiratory Distress Syndrome Requiring Extracorporeal Membrane Oxygenation^27^ | Petit et al. | 2022 | Mortality (at longest follow-up)  ECMO duration  MV duration  ICU LOS | ARDS pts on VV ECMO (n=91)  PP=38 |
| Prone position during ECMO is safe and improves oxygenation^28^ | Kipping et al. | 2013 | ECMO duration | ARDS pts on VV ECMO (n=12)  PP=12 |
| Prone positioning in severe ARDS requiring extracorporeal membrane oxygenation^29^ | Rilinger et al. | 2020 | Mortality (at longest follow-up)  ECMO duration  MV duration  ICU LOS | ARDS pts on VV ECMO (n=158)  PP=38 |
| Prone positioning during venovenous extracorporeal membrane oxygenation in acute respiratory distress syndrome: a multicentre cohort study and propensity-matched analysis^30^ | Giani et al. | 2021 | Mortality (at longest follow-up)  ECMO duration  ICU LOS | ARDS pts on VV ECMO (n=240)  PP=38 |
| Application of prone position in hypoxaemic patients supported by veno-venous ECMO^31^ | Lucchini et al. | 2018 | ECMO duration | ARDS pts on VV ECMO (n=14)  PP=14 |
| A single-centre study of safety and efficacy of prone positioning for critically ill patients on veno-venous extracorporeal membrane oxygenation^32^ | Chaplin et al. | 2021 | Long-term mortality (>90 days)  ECMO duration | ARDS pts on VV ECMO (n=72)  PP=13 |
| Efficacy of proning in acute respiratory distress syndrome on extracorporeal membrane oxygenation^33^ | Chang et al. | 2022 | ECMO duration  MV duration | ARDS (COVID-19) pts on VV ECMO (n=30)  PP=12 |

_MV – mechanical ventilation; ICU LOS-intensive care unit length of stay; pts- patients; PP- prone positioning_

Table 1. Summary of primary studies included in the updated MA for core question 3

| **Study** | **Selection (0–4)** | **Comparability (0–2)** | **Outcome (0–3)** | **Interpretation** |
| --- | --- | --- | --- | --- |
| **Chen et al., 2022** | ★★★★ | ★★ | ★★★ | Good quality |
| **Rilinger et al., 2020** | ★★★★ | ★★ | ★★★ | Good quality |
| **Massart et al., 2023** | ★★★★ | ★★ | ★★★ | Good quality |
| **Giani et al., 2021** | ★★★★ | ★★ | ★★★ | Good quality |
| **Petit et al., 2022** | ★★★★ | ★★ | ★★★ | Good quality |
| **Chaplin et al., 2021** | ★★★★ | - | ★★★ | Poor quality |
| **Garcia et al., 2020** | ★★★★ | - | ★★★ | Poor quality |
| **Chang et al., 2022** | ★★★★ | - | ★★★ | Poor quality |
| **Franchineau et al., 2020** | ★★★ | - | ★★★ | Poor quality |
| **Lucchini et al., 2018** | ★★★ | - | ★★★ | Poor quality |
| **Guervilly et al., 2019** | ★★★★ | ★★ | ★★★ | Good quality |
| **Kimmoun et al., 2015** | ★★★ | - | ★★★ | Poor quality |
| **Kipping et al., 2013** | ★★★ | - | ★★★ | Poor quality |
| **Zaaqoq et al., 2022** | ★★★★ | ★★ | ★★★ | Good quality |
| **Yang et al., 2021** | ★★★★ | - | ★★★ | Poor quality |

^Thresholds for converting the Newcastle-Ottawa scale to AHQR standards (good, fair and poor). Good quality: 3 or 4 stars in selection domain AND 1 or 2 stars in comparability domain AND 2 or 3 stars in outcome/exposure domain.^

^Fair quality: 2 stars in selection domain AND 1 or 2 stars in comparability domain AND 2 or 3 stars in outcome/exposure domain. Poor quality: 0 or 1 star in selection domain OR 0 stars in comparability domain OR 0 or 1 star in outcome/exposure domain.^

Table 2. NOS appraisal for cohort studies reviewed in the updated MA for core question 3

| **Study** | **D1** | **D2** | **D3** | **D4** | **D5** | **Overall** |
| --- | --- | --- | --- | --- | --- | --- |
| **Schmidt et al., 2023** | 🟢 | 🟢 | 🟢 | 🟢 | 🟢 | 🟢 |

^D1: bias arising from the randomisation from randomisation process D2: bias due to deviations from intended intervention D3: bias due to missing outcome data D4: bias in measurement of the outcome D5: bias in selection of the reported result^

Table 3. RoB 2.0 assessment of RCT reviewed in the updated MA for core question 3

Evidence Synthesis

For dichotomous outcomes, effect estimates were expressed as RR with 95% CI. For continuous outcomes, effect estimates were expressed as MD with 95% CI. Where data were missing or not directly reported, missing summary statistics were estimated from medians or interquartile ranges using established methods (Wan method^34^). Data was extracted from included studies using Covidence,^35^ RevMan^36^ and R^37^ software. Results were tabulated in summary tables or visually presented using forest plots. Pooled estimates were calculated using a random-effects model (DerSimonian and Laird method) to account for between-study heterogeneity. Statistical heterogeneity was quantified using the I^2^ statistic and between-study variance (τ ^2^).

| **Demographic** | **All** | **Prone** | **No prone** |
| --- | --- | --- | --- |
| Age | 49.5 (45.8-53.2) | 49.1 (45.6-51.6) | 49.5 (47.1-51.9) |
| Male | 68.1% (65.3%-70.8%) | 69.1% (64.8%-73.1%) | 68.0% (63.4%-72.3%) |

Table 4. Baseline Demographics


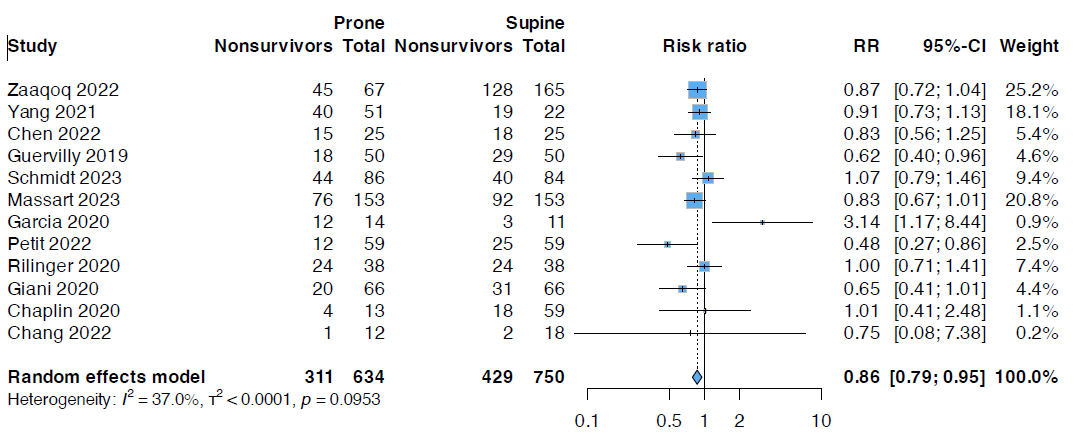


Figure 3. Forest plot outcome: Mortality (assessed as Risk Ratio)


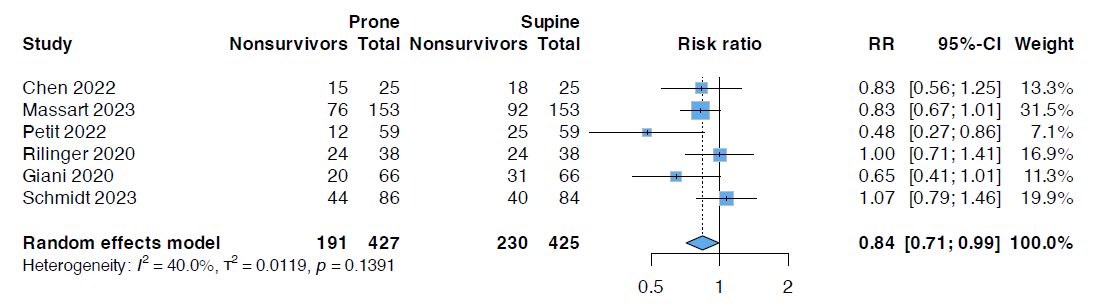


Figure 4. Forest plot comparison: propensity score-matched and RCT studies


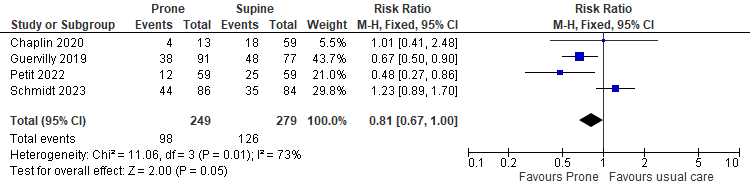


Figure 5. Forest plot outcome: Long-term mortality (≥90D)

| Outcome | Group | Estimate | LCI | UCI |
| --- | --- | --- | --- | --- |
| ECMO duration | Prone position (mean) | 18.8 days | 15.4 | 22.2 |
|  | When compared to usual care alone  P=0.0003 | +7.1 days | +3.2 | +10.9 |
| ICU LOS | Prone position (mean) | 40.2 days | 31.5 | 48.8 |
|  | When compared to usual care alone  P=0.027 | +7.6 days | +0.9 | +14.4 |

Table 5. Secondary outcomes when comparing between prone position versus usual care alone during VV ECMO

Figure 5. Effect of prone positioning in ARDS patients on VV ECMO on (i) median static compliance (ii) median P:F ratio (iii) median MV duration

Sensitivity analyses were performed to assess the robustness of the pooled mortality estimate (44.5% [95% CI 34.4 to 56.7]). Results were consistent across the application of the random-effects trim-and-fill procedure (49.6% [CI 95% 37.1 to 62.1]), and exclusion of studies judged to be at high risk of bias (43.4% [95% CI 34.7 to 53.7]).


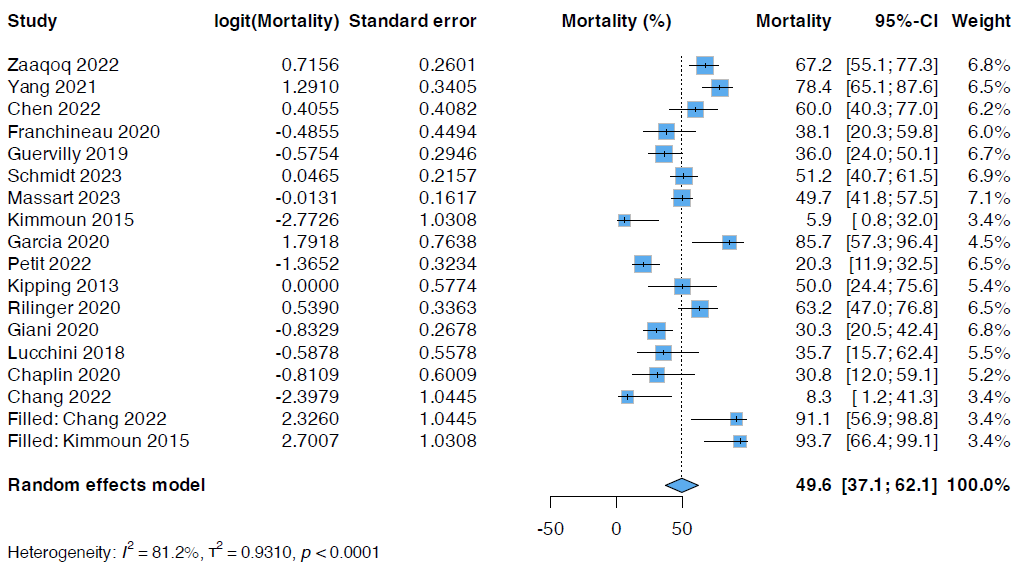


Figure 6. Forest Plot outcome: Mortality post-trim-and fill

Meta-regression was performed to investigate whether study-level covariates influenced the effect estimate for mortality. Duration of ECMO demonstrated a statistically significant (p=0.033) negative association, suggesting that longer ECMO runs were linked with a smaller treatment effect of prone positioning. Arterial pH was significantly associated with lower baseline pH values correlating with worse outcomes (p= 0.0063). These results suggest that disease severity indicators may partially account for heterogeneity in study outcomes, whereas demographic factors and ventilatory settings contributed little explanatory power.

| Covariate | Study | Regression coefficient | Lower CI | Upper CI | P Value |
| --- | --- | --- | --- | --- | --- |
| Age | 14 | 0.0382 | -0.0415 | 0.118 | 0.35 |
| BMI | 7 | 0.398 | -0.162 | 0.968 | 0.16 |
| **Duration of ECMO** | **14** | **-0.050** | **-0.096** | **-0.004** | **0.033** |
| Time to proning | 8 | -0.16 | -0.34 | 0.01 | 0.070 |
| Compliance | 6 | -0.010 | -0.45 | 0.44 | 0.97 |
| PF ratio | 9 | -0.004 | -0.025 | 0.018 | 0.72 |
| **pH** | **6** | **-66.23** | **-113.77** | **-18.71** | **0.0063** |
| PEEP | 7 | 0.093 | -0.23 | 0.42 | 0.58 |

^*Covariates with p<0.05 have been highlighted in bold 95% CI intervals and associated^ *^p^* ^values; BMI: body mass index;^

Table 6. Table of study-level covariates

For the primary outcome of mortality at the longest reported follow-up, a trial sequential analysis (TSA) was performed for propensity score-matched observational studies and RCTs to determine whether the available evidence reached the required OIS to confirm a robust effect. The required OIS was estimated at 1702 patients, meaning that only when the accrued sample size reaches this threshold can a conventional significance level of *p<0.05* be interpreted as conclusive in the TSA framework. The cumulative Z-curve crossed the conventional significance boundary (*p<0.05*), consistent with the MA result suggesting a mortality benefit. However, the Z-curve did not cross the TSA-adjusted boundary for benefit, indicating that the current evidence is insufficient to confirm a definitive effect and remains at risk of type I error. Similarly, the futility boundary was not crossed, meaning the available data do not exclude the possibility of a clinically meaningful difference.


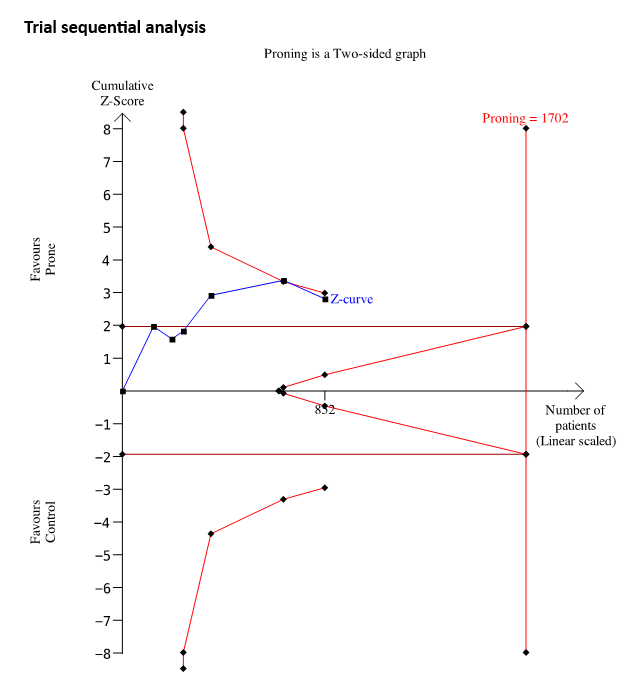


^Blue line represents the cumulative Z-curve of the pooled studies; Horizontal dark red lines represent conventional significance boundaries (^*^p^* ^<0.05); Light red curves show TSA-adjusted monitoring boundaries for benefit and harm; Inner triangular region denotes futility boundary;^

Figure 7. TSA analysis of prone positioning during VV-ECMO for ARDS

Subgroup analyses were prespecified to examine potential effect modifiers, including study design and are shown in the table below.

| Analysis | Subgroup | Estimate |
| --- | --- | --- |
| Aetiology of ARDS  P=0.05 | COVID-19 | 59.7% (40.4%-76.5%) |
|  | Non-COVID-19 | 37.4% (27.4%-48.6%) |
| Study type  P=0.40 | Unadjusted | 45.2% (25.6%-66.5%) |
|  | Matched without propensity score | 36.0% (24.0%-50.1%) |
|  | Propensity score matched | 43.4% (27.5%-60.7%) |
|  | RCT | 51.2% (40.7%-61.5%) |

Table 7. Prespecified Sub-group analyses

Certainty of evidence

The quality of included evidence was assessed using the GRADE framework and shown in the SoF table below.

Considerable clinical and methodological heterogeneity was observed across the included studies, likely contributing to variability in effect estimates. First, the patient population differed substantially, with some studies enrolling only COVID-19-related ARDS, potentially influencing the generalisability of these results. Second, the timing of prone positioning in relation to ECMO initiation varied widely, ranging from early standardised protocols (*e.g.* within 48 hrs of ECMO) to non-standardised or clinician-discretion approaches. Third, the dose or duration of prone positioning sessions was inconsistently reported and applied; most studies aimed for sessions of 16 to 18 hrs, several did not specify duration, limiting comparability.

Overall, the certainty of evidence was rated very low, primarily due to limitations in study design and risk of bias. Of the included studies, only one was a RCT (PRONECMO), which was methodologically robust. The remaining studies were non-randomised studies rated poor to good quality using the thresholds for converting the NOS to AHRQ standards. This was largely due to serious concerns regarding time-varying confounders such as disease severity at the time of prone positioning and the timing of ECMO initiation, as well as non-random allocation of the intervention, which introduces the potential for selection bias and residual confounding despite statistical adjustment.

Despite these methodological limitations, the decision was made not to downgrade for imprecision in the primary outcome of mortality at the longest follow-up. This decision was based on the observation that the cumulative sample size across studies exceeded the OIS, and that the confidence intervals around the pooled estimate did not cross thresholds for both benefit and harm. Point estimates were consistent across studies, and subgroup analyses all produced directionally similar results. Additionally, the TSA supported the adequacy of accrued information, reinforcing the decision not to downgrade.

Although the TSA suggests that while prone positioning during VV ECMO for ARDS may be associated with improved survival, the current cumulative evidence remains underpowered, and further studies are required to reach firm conclusions. The sole RCT included in this analysis demonstrated no significant difference between groups (prone position versus supine position) in the primary outcome of successful weaning of ECMO within 60 days of randomisation (RD 0.1% [CI -14.9% to 15.2%]) and no significant differences in any prespecified secondary endpoints.^23^ Of note, the majority of PRONECMO population had COVID-19-related ARDS (93.5%), which raises concerns regarding the generalisability to patients with non-COVID-19-related ARDS.

In addition to the outcomes formally assessed in the GRADE SoF, we sought to illustrate several relevant physiological and safety outcomes reported across the included studies. These were considered important to provide a more comprehensive picture of the effects of prone positioning during VV ECMO, despite the significant heterogeneity, small sample sizes and descriptive nature of reporting.

Across six studies, prone positioning during VV ECMO demonstrated variable improvements in oxygenation, as reflected by the P:F ratio, as illustrated in Figure 9. ^20,25,26,28,31,32^ However, the general trend suggests a modest improvement in oxygenation parameters. Three studies reported changes in static compliance before and after prone positioning (Figure 8).^21,25,32^ Overall, prone positioning was potentially associated with modest improvements in compliance, however, the effect is not uniform across patient populations. Two studies reported cardiac arrest events and showed prone positioning during VV ECMO reduced the incidents (RR 0.45 [CI 0.19 to 1.05]).^23,27^

Several studies reported clinically relevant complications during prone positioning, including a reduction in ECMO flow 16/224 patients (7.14%),^25,26,30^ desaturation events 17/267 patients (6.36%),^20,28,30^ and haemodynamic instability 10/267 patients (3.74%).^20,28,30^ These outcomes were presented to the GDG to provide context on the potential physiological benefits and risks of prone positioning in this population. While they were not subjected to formal GRADE assessment, their inclusion highlights key aspects of safety and patient-centred considerations that may inform the evidence-to-decision process.

Evidence to Decision Framework

The GDG made a judgment about the direction and strength of the recommendation based on the certainty of evidence, integrating other critical considerations beyond effect estimates.

ARDS requiring VV ECMO represents a critical illness with high mortality and significant resource use. Interventions that may improve outcomes in this population are of high clinical importance and were prioritised by the GDG.

Across eleven studies (ten non-randomised and one RCT), prone positioning during VV ECMO was associated with a reduction in mortality (RR 0.86 [CI 95% 0.78 to 0.95]), corresponding to 76 fewer deaths per 1000 patients. Although rated as very low certainty due to study design limitations and risk of bias, the GDG considered these potential benefits clinically meaningful.

Prone positioning was also associated with increased ECMO duration (MD 7.1 days [CI 95% 3.2 to 10.9]) and ICU length of stay (MD 7.6 days [CI 95% 0.9 to 14.4]). These findings were not linked to excessive adverse events, suggesting that longer duration of support may reflect prolonged survival rather than harm. Nonetheless, such extensions could increase healthcare utilisation. Importantly, the impact of longer ICU or ECMO duration on long-term quality of life was not evaluated in the included studies and remains uncertain.

The GDG did not conduct a de novo systematic review to specifically identify studies reporting critical outcomes not captured in the included MA. It was considered highly unlikely that primary studies would have reported such outcomes without also reporting short-term mortality. Therefore, it was decided that conducting an additional review was unlikely to yield new eligible studies.

The GDG judged that there is likely little variation in how patients and clinicians value mortality outcomes. Survival is universally prioritised in this setting and longer ICU or ECMO duration is often viewed as acceptable if associated with improved outcomes. However, patients may place differing value on potential long-term physical and psychological impacts of critical illness, which remain underexplored in this population. Evidence from core outcome sets developed for critically ill patients receiving ECMO supports this interpretation. Hodgson et al. used a rigorous, systematic process, including the modified Delphi method, with input from patients, families, clinicians and researchers to identify the most critical outcomes.^38,39^ Core outcomes selected fell into three domains: adverse events, death and life impact. The outcomes chosen for this PICO are encompassed within these domains.

The principal concern with implementing prone positioning during VV ECMO is its resource intensity. No formal cost-effectiveness analyses were available; however, a mortality benefit, if confirmed, may justify the additional resource demands in high-resource centres. The feasibility of prone positioning during VV ECMO is well described;^20,26,30,32^ however, its implementation is largely restricted to experienced centres with staff who are specifically trained in performing the procedure.^18,27,28,30^ The procedure requires skilled personnel, training and multidisciplinary coordination. Longer duration of support in less-resourced settings may further increase cost and strain system capacity. The primary barriers identified in the literature included a lack of training and expertise, as well as patient safety concerns.^33^

There are no studies directly evaluating the resource utilisation of prone positioning during VV ECMO. Feasibility and acceptability are greater in centres with established prone positioning protocols and ECMO experience. In contrast, implementation may be challenging in less-resourced or lower-volume centres, potentially exacerbating health inequities. Despite these challenges, prone positioning is a well-established intervention in ARDS management and is generally accepted among clinicians familiar with ECMO practice.

|  | **Judgement** | | | | | | |
| --- | --- | --- | --- | --- | --- | --- | --- |
| **Problem** | No | Probably no | **Probably yes** | Yes |  | Varies | Don't know |
| **Desirable Effects** | Trivial | Small | Moderate | Large |  | **Varies** | Don't know |
| **Undesirable Effects** | Large | Moderate | **Small** | Trivial |  | Varies | Don't know |
| **Certainty of evidence** | **Very low** | Low | Moderate | High |  |  | No included studies |
| **Values** | Important uncertainty or variability | Possibly important uncertainty or variability | **Probably no important uncertainty or variability** | No important uncertainty or variability |  |  |  |
| **Balance of effects** | Favors the comparison | **Probably favors the comparison** | Does not favor either the intervention or the comparison | Probably favors the intervention | Favors the intervention | Varies | Don't know |
| **Resources required** | Large costs | **Moderate costs** | Negligible costs and savings | Moderate savings | Large savings | Varies | Don't know |
| **Certainty of evidence of required resources** | Very low | Low | Moderate | High |  |  | **No included studies** |
| **Cost effectiveness** | Favors the comparison | Probably favors the comparison | Does not favor either the intervention or the comparison | Probably favors the intervention | Favors the intervention | Varies | **No included studies** |
| **Equity** | Reduced | Probably reduced | **Probably no impact** | Probably increased | Increased | Varies | Don't know |
| **Acceptability** | No | Probably no | **Probably yes** | Yes |  | Varies | Don't know |
| **Feasibility** | No | Probably no | **Probably yes** | Yes |  | Varies | Don't know |

Table 8. EtD Table of Judgement for core question 3

Subgroup considerations

Several potential subgroups may be important to consider when evaluating the effects of prone positioning during VV ECMO in patients with ARDS. While most included studies did not report stratified outcomes, some observational data suggest that the effects of prone positioning during VV ECMO may vary by timing of the intervention, severity of lung injury or underlying cause of ARDS. Earlier initiation of prone positioning may be associated with greater mortality benefit. Feasibility and safety concerns may limit use in patients with obesity or haemodynamic instability. Future prospective trials should predefine and report subgroup effects to better inform clinical decisions.

Future research priorities

The PRONECMO trial provides moderate-certainty evidence suggesting that prone positioning during VV ECMO does not improve outcomes in patients with severe ARDS. However, significant questions remain regarding generalisability, optimal timing and specific patient subgroups (non-COVID ARDS, severe vs moderate hypoxaemia, high vs low compliance lungs). High-quality RCTs including patients with a diverse ARDS population would better define efficacy. They should specify a standardised protocol for the intervention, to eliminate variability in the timing, frequency and duration of prone positioning across centres. Registry data can be more efficiently utilised to minimise bias and confounding with propensity score techniques. Long-term patient-centred outcomes should include functional status, quality of life and neurocognitive recovery.

| **Summary of findings:** | | | | | | |
| --- | --- | --- | --- | --- | --- | --- |
| **Prone positioning compared to usual care in ARDS patients receiving VV ECMO** | | | | | | |
| **Patient or population:** ARDS patients receiving VV ECMO  **Setting:** Intensive Care Units across Australia  **Intervention:** prone positioning  **Comparison:** usual care | | | | | | |
| Outcomes | **Anticipated absolute effects^*^** (95% CI) | | Relative effect (95% CI) | № of participants (studies) | Certainty of the evidence (GRADE) | Comments |
|  | **Risk with usual care** | **Risk with prone positioning** |  |  |  |  |
| Mortality (at longest follow up) assessed with: Risk Ratio | 541 per 1,000 | **465 per 1,000** (422 to 514) | **RR 0.86** (0.78 to 0.95) | 852 (6 non-randomised studies)^1,2,3,4,5,6^ | ⨁◯◯◯ Very low^a^ | Prone positioning may reduce mortality (at longest follow-up) but the evidence is very uncertain. |
| Long term mortality (>/=90 days) | 452 per 1,000 | **366 per 1,000** (303 to 452) | **RR 0.81** (0.67 to 1.00) | 528 (4 non-randomised studies)^2,6,7,8^ | ⨁◯◯◯ Very low^a^ | Prone positioning may reduce long-term mortality (>/=90 days) but the evidence is very uncertain. |
| ECMO duration assessed with: days | The mean ECMO duration was **0** days | MD **7.1 days more** (3.2 more to 10.9 more) | - | 1079 (10 non-randomised studies)^1,2,3,4,5,6,7,8,9,10^ | ⨁◯◯◯ Very low^a^ | Prone positioning may increase ECMO duration but the evidence is very uncertain. |
| ICU length of stay assessed with: days | The mean ICU length of stay was **0** days | MD **7.6 days more** (0.9 more to 14.4 more) | - | 514 (5 non-randomised studies)^1,2,3,5,6^ | ⨁◯◯◯ Very low^a^ | Prone positioning may increase ICU length of stay but the evidence is very uncertain. |
| ***The risk in the intervention group** (and its 95% confidence interval) is based on the assumed risk in the comparison group and the **relative effect** of the intervention (and its 95% CI).  **CI:** confidence interval; **MD:** mean difference; **RR:** risk ratio | | | | | | |
| **GRADE Working Group grades of evidence** **High certainty:** we are very confident that the true effect lies close to that of the estimate of the effect. **Moderate certainty:** we are moderately confident in the effect estimate: the true effect is likely to be close to the estimate of the effect, but there is a possibility that it is substantially different. **Low certainty:** our confidence in the effect estimate is limited: the true effect may be substantially different from the estimate of the effect. **Very low certainty:** we have very little confidence in the effect estimate: the true effect is likely to be substantially different from the estimate of effect. | | | | | | |

#### Explanations

#### a. serious concerns regarding time-varying confounders (such as disease progression severity and time of proning) and non-random allocation of proning

**Prognosis: What is the course of survival in ARDS over VV ECMO alone**

| **№ of studies** | **Certainty assessment** | | | | | | **Effect** | | | **Certainty** | **Importance** |
| --- | --- | --- | --- | --- | --- | --- | --- | --- | --- | --- | --- |
|  | **Study design** | **Risk of bias** | **Inconsistency** | **Indirectness** | **Imprecision** | **Other considerations** | **№ of events** | **№ of individuals** | **Rate (95% CI)** |  |  |
| Mortality (assessed with: %) | | | | | | | | | | | |
| 16^1,2,3,4,5,6,7,8,9,10,11,12,13,14,15^ | non-randomised studies | not serious | not serious^a^ | not serious | not serious | none | 331 | 698 | event rate 45.4 per 100 (34.4 to 57) | ⨁⨁⨁⨁ High^a^ | CRITICAL |
| Duration of ECMO (assessed with: days) | | | | | | | | | | | |
| 14^2,4,5,6,7,8,9,10,11,12,13,14,15,16^ | non-randomised studies | not serious | not serious | not serious | not serious | none | - | 580 | mean 18.8 days (15.4 to 22.2) | ⨁⨁⨁⨁ High | IMPORTANT |
| ICU Length of Stay (assessed with: days) | | | | | | | | | | | |
| 9^2,4,5,6,7,8,10,11,15^ | non-randomised studies | not serious | not serious | not serious | not serious | none | - | 322 | mean 40.2 days (31.5 to 48.8) | ⨁⨁⨁⨁ High | IMPORTANT |

Certainty ratings for prognostic questions were based on the GRADE approach for prognosis, where observational evidence begins as high certainty, and may be rated down or up based on predefined criteria.

#### Explanations

a. borderline decision to not downgrade for inconsistency. high I2 values and confidence intervals sometimes overlapped. However, inconsistency was accounted for based on subgroup and meta-analyses

References

1. International Guidelines Library [Internet]. GIN. [cited 2023 Feb 20]. Available from: https://g-i-n.net/international-guidelines-library

2. ECRI Guidelines Trust® [Internet]. ECRI. [cited 2023 Feb 20]. Available from: https://d84vr99712pyz.cloudfront.net/p/images1/ecri-trusted-voice-healthcare.jpg

3. EtD’s and Guidelines [Internet]. [cited 2023 Feb 20]. Available from: https://guidelines.gradepro.org/search

4. MAGICapp - Making GRADE the Irresistible Choice - Guidelines and Evidence summaries [Internet]. [cited 2023 Feb 20]. Available from: https://app.magicapp.org/#/guidelines

5. BIGG-REC - GRADE Recommendations [Internet]. [cited 2023 Feb 20]. Available from: https://bigg-rec.bvsalud.org/en

6. Find guidance [Internet]. NICE. NICE; [cited 2023 Feb 20]. Available from: https://www.nice.org.uk/guidance

7. ELSO Guidelines | Extracorporeal Membrane Oxygenation (ECMO) [Internet]. [cited 2023 Feb 20]. Available from: https://www.elso.org/ecmo-resources/elso-ecmo-guidelines.aspx

8. Trip Medical Database [Internet]. [cited 2023 Feb 20]. Available from: https://www.tripdatabase.com/

9. Epistemonikos: Database of the best Evidence-Based Health Care [Internet]. [cited 2023 Feb 20]. Available from: https://www.epistemonikos.org/

10. PROSPERO: International prospective register of systematic reviews [Internet]. PROSPERO. [cited 2023 Feb 20]. Available from: https://www.crd.york.ac.uk/prospero/

11. Poon WH, Ramanathan K, Ling RR, Yang IX, Tan CS, Schmidt M, et al. Prone positioning during venovenous extracorporeal membrane oxygenation for acute respiratory distress syndrome: a systematic review and meta-analysis. Crit Care Lond Engl. 2021 Aug 12;25(1):292.

12. PubMed [Internet]. PubMed. [cited 2024 Sept 13]. Available from: https://pubmed.ncbi.nlm.nih.gov/

13. Search | Cochrane Library [Internet]. [cited 2023 Feb 20]. Available from: https://www.cochranelibrary.com/central

14. Welcome - Embase [Internet]. [cited 2023 Feb 20]. Available from: https://www.embase.com/landing?status=grey

15. Scopus - Homepage [Internet]. [cited 2025 Aug 14]. Available from: https://www-scopus-com.wwwproxy1.library.unsw.edu.au/pages/home?display=basic#basic

16. Sterne JAC, Savović J, Page MJ, Elbers RG, Blencowe NS, Boutron I, et al. RoB 2: a revised tool for assessing risk of bias in randomised trials. BMJ. 2019 Aug 28;366:l4898.

17. Ottawa Hospital Research Institute [Internet]. [cited 2025 Aug 25]. Available from: https://www.ohri.ca/programs/clinical_epidemiology/oxford.asp

18. Zaaqoq AM. Beneficial Effect of Prone Positioning During Venovenous Extracorporeal Membrane Oxygenation for Coronavirus Disease 2019*. Crit Care Med. 2022 Feb;Volume 50(Number 2):275–85.

19. Yang X, Hu M, Yu Y, Zhang X, Fang M, Lian Y, et al. Extracorporeal Membrane Oxygenation for SARS-CoV-2 Acute Respiratory Distress Syndrome: A Retrospective Study From Hubei, China. Front Med. 2021 Jan 12;7:611460.

20. Chen Z, Li M, Gu S, Huang X, Xia J, Ye Q, et al. Effect of prone position in patients with acute respiratory distress syndrome supported by venovenous extracorporeal membrane oxygenation: a retrospective cohort study. BMC Pulm Med. 2022 Dec;22(1):234.

21. Franchineau G, Bréchot N, Hekimian G, Lebreton G, Bourcier S, Demondion P, et al. Prone positioning monitored by electrical impedance tomography in patients with severe acute respiratory distress syndrome on veno-venous ECMO. Ann Intensive Care. 2020 Dec;10(1):12.

22. Guervilly C, Prud’homme E, Pauly V, Bourenne J, Hraiech S, Daviet F, et al. Prone positioning and extracorporeal membrane oxygenation for severe acute respiratory distress syndrome: time for a randomized trial? Intensive Care Med. 2019 July;45(7):1040–2.

23. Schmidt M, Hajage D, Lebreton G, Dres M, Guervilly C, Richard JC, et al. Prone Positioning During Extracorporeal Membrane Oxygenation in Patients With Severe ARDS: The PRONECMO Randomized Clinical Trial. JAMA. 2023 Dec 26;330(24):2343.

24. Massart N, Guervilly C, Mansour A, Porto A, Flécher E, Esvan M, et al. Impact of Prone Position in COVID-19 Patients on Extracorporeal Membrane Oxygenation*. Crit Care Med. 2023 Jan;51(1):36–46.

25. Kimmoun A, Roche S, Bridey C, Vanhuyse F, Fay R, Girerd N, et al. Prolonged prone positioning under VV-ECMO is safe and improves oxygenation and respiratory compliance. Ann Intensive Care. 2015 Dec;5(1):35.

26. Garcia B, Cousin N, Bourel C, Jourdain M, Poissy J, Duburcq T, et al. Prone positioning under VV-ECMO in SARS-CoV-2-induced acute respiratory distress syndrome. Crit Care. 2020 Dec;24(1):428.

27. Petit M, Fetita C, Gaudemer A, Treluyer L, Lebreton G, Franchineau G, et al. Prone-Positioning for Severe Acute Respiratory Distress Syndrome Requiring Extracorporeal Membrane Oxygenation. Crit Care Med. 2022 Feb;50(2):264–74.

28. Kipping V, Weber-Carstens S, Lojewski C, Feldmann P, Rydlewski A, Boemke W, et al. Prone Position during ECMO is Safe and Improves Oxygenation. Int J Artif Organs. 2013 Nov;36(11):821–32.

29. Rilinger J, Zotzmann V, Bemtgen X, Schumacher C, Biever PM, Duerschmied D, et al. Prone positioning in severe ARDS requiring extracorporeal membrane oxygenation. Crit Care. 2020 Dec;24(1):397.

30. Giani M, Martucci G, Madotto F, Belliato M, Fanelli V, Garofalo E, et al. Prone Positioning during Venovenous Extracorporeal Membrane Oxygenation in Acute Respiratory Distress Syndrome. A Multicenter Cohort Study and Propensity-matched Analysis. Ann Am Thorac Soc. 2021 Mar;18(3):495–501.

31. Lucchini A, De Felippis C, Pelucchi G, Grasselli G, Patroniti N, Castagna L, et al. Application of prone position in hypoxaemic patients supported by veno-venous ECMO. Intensive Crit Care Nurs. 2018 Oct;48:61–8.

32. Chaplin H, McGuinness S, Parke R. A single-centre study of safety and efficacy of prone positioning for critically ill patients on veno-venous extracorporeal membrane oxygenation. Aust Crit Care. 2021 Sept;34(5):446–51.

33. Chang SH, Smith DE, Carillo JA, Sommer PM, Geraci TC, Williams D, et al. Efficacy of proning in acute respiratory distress syndrome on extracorporeal membrane oxygenation. JTCVS Tech. 2022 Dec;16:109–16.

34. Wan X, Wang W, Liu J, Tong T. Estimating the sample mean and standard deviation from the sample size, median, range and/or interquartile range. BMC Med Res Methodol. 2014 Dec 19;14(1):135.

35. Covidence - Better systematic review management [Internet]. [cited 2024 Aug 28]. Available from: https://www.covidence.org/

36. The Cochrane Collaboration, 2020. Review Manager (RevMan).

37. R: The R Project for Statistical Computing [Internet]. [cited 2025 Oct 23]. Available from: https://www.r-project.org/

38. Hodgson CL, Burrell AJC, Engeler DM, Pellegrino VA, Brodie D, Fan E, et al. Core Outcome Measures for Research in Critically Ill Patients Receiving Extracorporeal Membrane Oxygenation for Acute Respiratory or Cardiac Failure: An International, Multidisciplinary, Modified Delphi Consensus Study. Crit Care Med. 2019 Nov;47(11):1557–63.

39. Hodgson CL, Fulcher B, Mariajoseph FP, Burrell AJC, Pellegrino V, Brodie D, et al. A Core Outcome Set for Research in Patients on Extracorporeal Membrane Oxygenation. Crit Care Med. 2021 Dec 1;49(12):e1252–4.
